# COnstrained Reference frame diffusion TEnsor Correlation Spectroscopic (CORTECS) MRI: A practical framework for high-resolution diffusion tensor distribution imaging

**DOI:** 10.3389/fnins.2022.1054509

**Published:** 2022-12-15

**Authors:** Alexandru V. Avram, Kadharbatcha S. Saleem, Peter J. Basser

**Affiliations:** ^1^Eunice Kennedy Shriver National Institute of Child Health and Human Development, National Institutes of Health, Bethesda, MD, United States; ^2^Center for Neuroscience and Regenerative Medicine, Bethesda, MD, United States; ^3^Henry M. Jackson Foundation for the Advancement of Military Medicine Inc., Bethesda, MD, United States

**Keywords:** diffusion tensor distribution (DTD), multidimensional diffusion MRI, microscopic anisotropy, cortical cytoarchitecture, microscopic principal diffusivity, diffusion reference frame, high-resolution diffusion MRI, multiple diffusion encoding

## Abstract

High-resolution imaging studies have consistently shown that in cortical tissue water diffuses preferentially along radial and tangential orientations with respect to the cortical surface, in agreement with histology. These dominant orientations do not change significantly even if the relative contributions from microscopic water pools to the net voxel signal vary across experiments that use different diffusion times, *b*-values, TEs, and TRs. With this in mind, we propose a practical new framework for imaging non-parametric diffusion tensor distributions (DTDs) by constraining the microscopic diffusion tensors of the DTD to be diagonalized using the same orthonormal reference frame of the mesoscopic voxel. In each voxel, the constrained DTD (cDTD) is completely determined by the correlation spectrum of the microscopic principal diffusivities associated with the axes of the voxel reference frame. Consequently, all cDTDs are inherently limited to the domain of positive definite tensors and can be reconstructed efficiently using Inverse Laplace Transform methods. Moreover, the cDTD reconstruction can be performed using only data acquired efficiently with single diffusion encoding, although it also supports datasets with multiple diffusion encoding. In tissues with a well-defined architecture, such as the cortex, we can further constrain the cDTD to contain only cylindrically symmetric diffusion tensors and measure the 2D correlation spectra of principal diffusivities along the radial and tangential orientation with respect to the cortical surface. To demonstrate this framework, we perform numerical simulations and analyze high-resolution dMRI data from a fixed macaque monkey brain. We estimate 2D cDTDs in the cortex and derive, in each voxel, the marginal distributions of the microscopic principal diffusivities, the corresponding distributions of the microscopic fractional anisotropies and mean diffusivities along with their 2D correlation spectra to quantify the cDTD shape-size characteristics. Signal components corresponding to specific bands in these cDTD-derived spectra show high specificity to cortical laminar structures observed with histology. Our framework drastically simplifies the measurement of non-parametric DTDs in high-resolution datasets with mesoscopic voxel sizes much smaller than the radius of curvature of the underlying anatomy, e.g., cortical surface, and can be applied retrospectively to analyze existing diffusion MRI data from fixed cortical tissues.

## 1. Introduction

By quantifying the microscopic motions of water molecules diffusion MRI (dMRI) provides a sensitive clinical tool to non-invasively probe the tissue structures at length scales (≈5*μm*) much smaller than the voxel size. In isotropic and anisotropic tissues, the dMRI signal at low diffusion sensitizations (*b*-values) can be described phenomenologically using diffusion tensor imaging (DTI) (Basser et al., 1994a,b). In DTI, the diffusion signal attenuation in each voxel is modeled using a diffusion tensor, **D**, which has 6 degrees of freedom. The diffusion tensor can be decomposed or diagonalized in an orthogonal reference frame whose principal coordinate axes are characterized by the eigenvectors ϵ**_1_**, ϵ**_2_**, *and* ϵ**_3_**. The normalized orthogonal unit vectors along the principal tensor axes represent 3 degrees of freedom of **D** that define its orientation with respect to the laboratory reference frame. The scalar principal diffusivities λ_1_, λ_2_, λ_3_ corresponding to these directions represent the other 3 degrees of freedom of **D** and determine the mean diffusivity and diffusion anisotropy. In general, **D** can be written as:


(1) $$\begin{array}{l} \text{D}=\lambda_{1}\epsilon_{1}\epsilon_{1}^{T}+\lambda_{2}\epsilon_{2}\epsilon_{2}^{T}+\lambda_{3}\epsilon_{3}\epsilon_{3}^{T}, \end{array}$$

where $\epsilon_{1}{\epsilon_{1}}^{T},\epsilon_{2}{\epsilon_{2}}^{T},\epsilon_{3}{\epsilon_{3}}^{T}$ are the principal coordinate axes dyads (or rank-1 tensors) derived from the eigenvectors of the diffusion tensor while the positivity of the principal diffusivities (i.e., eigenvalues of **D**) guarantees that **D** is positive definite.

However, at *b*-values larger than 1, 500*s*/*mm*^2^ the dMRI tissue signal is more sensitive to the intravoxel variation of water diffusion properties, and the DTI approximation may no longer hold. To quantify the intravoxel diffusion heterogeneity many approaches have been proposed, including using signal representations with higher-order terms, such as diffusion kurtosis imaging (DKI) (Jensen et al., 2005), generalized diffusion tensor imaging (GDTI) (Özarslan and Mareci, 2003; Liu et al., 2004), mean apparent propagator (MAP) MRI (Özarslan et al., 2013; Avram et al., 2016), as well as multi-exponential, multi-tensor, or multi-compartment tissue diffusion models (Stanisz et al., 1997; Mulkern et al., 1999; Assaf and Basser, 2005; Zhang et al., 2012).

Jian et al., extended the multi-tensor signal representations to describe intravoxel diffusion heterogeneity using a Wishart distribution of microscopic diffusion tensors (Jian et al., 2007). Even though this parametric distribution is limited in its ability to accurately quantify the range of diffusion heterogeneity in healthy and diseased tissues, it nonetheless inspired great interest in measuring subvoxel distributions of microscopic diffusion tensors (DTDs). In general, however, to disentangle microscopic processes with arbitrary diffusivities, diffusion anisotropies, and orientations, it is necessary to sensitize the measurement to diffusion-diffusion correlations (Cory et al., 1990; Mitra, 1995; Callaghan and Komlosh, 2002) by preparing the signal with multiple pulsed-field gradients (mPFG), or multiple diffusion encodings (MDE). Historically, biological and clinical applications of mPFG or MDE methods (Komlosh et al., 2007) have focused on estimating microstructural parameters such as the average axon diameters (Koch and Finsterbusch, 2008; Avram et al., 2013a,b; Komlosh et al., 2018) or pore size distributions (Benjamini et al., 2016). More recently, MDE-prepared MRI measurements were described using tensor-valued diffusion encoding (Westin et al., 2016; Topgaard, 2017) in the context of probing subvoxel diffusion heterogeneity described using an ensemble of non-exchanging Gaussian diffusion tensor processes whose corresponding ellipsoids have distinct sizes, shapes, and orientations, i.e., the DTD.

While, at least in principle, one can reconstruct DTDs from a very large number of measurements with encodings sampling the 6D space of b-tensors, in practice, the limited signal-to-noise ratio (SNR) and long scan duration make such clinical or biological experiments very challenging (Topgaard, 2017; Song et al., 2022). To reduce the requirements for the high SNR level and a large number of measurements some have made simplifying assumptions such as cylindrical symmetry of microscopic tensors (Topgaard, 2017) which reduce the dimensionality of non-parametric DTD reconstructions from six to four degrees of freedom. Alternatively, one can use parametric models (e.g., analytical functions) to estimate features of the DTDs (Jian et al., 2007; Lasic et al., 2014; Szczepankiewicz et al., 2016; Westin et al., 2016; Magdoom et al., 2021) from data acquired using MDE and conventional single diffusion encoding (SDE) (Stejskal and Tanner, 1965).

Meanwhile, numerous studies using dMRI and other modalities provide converging evidence that, at a sufficiently small (i.e., mesoscopic) length scale, neuronal tissues, including cortical gray matter (GM) are organized preferentially along local orthogonal frames of reference. Ever since the earliest observations of cortical cyto- and myeloarchitecture (Brodmann, 1909; Cajal, 1909; Vogt, 1910), histochemistry and immunohistochemistry studies have consistently shown that cellular and subcellular structures at the microscopic scale are oriented predominantly along orthogonal, i.e., radial and tangential, orientations with respect to the cortical surface. This orthogonal reference frame persists at larger, mesoscopic scales of tens and hundreds of micrometers, and can be clearly seen in the arrangements of cells with various sizes, shapes, and densities forming tissue architectural patterns along the same radial and tangential orientations such as cortical columns and laminae, respectively (Amunts and Zilles, 2015; Rubenstein and Rakic, 2020). Most recently, studies using state-of-the-art electron microscopy (EM) in cortical GM (Lichtman and Denk, 2011; Shapson-Coe et al., 2021) have mapped the 3D organization of neuronal cells in gray matter with nanometer resolution over fields-of-view (FOVs) of hundreds of micrometers. These studies revealed in unprecedented detail anisotropic tissue structures, such as the microvasculature (Zhang et al., 2015), branching dendrites, neurofilaments, and other cell processes in various neuronal and non-neuronal cells (pyramidal neurons, intrinsic neurons, glial cells, etc.) roughly aligned along a local orthogonal frame of reference.

At mesoscopic length scales of a few 100 *μ*m, diffusion processes in neural tissues align closely with the dominant orientations in the local tissue microstructure. Histological validation studies using ultra high-resolution dMRI have consistently found a good correspondence between the orientations of the underlying tissue microstructure and the orthogonal DTI reference frame (Budde and Annese, 2013; Seehaus et al., 2013, 2015) defined by $\epsilon_{1}\epsilon_{1}{\;}^{T},\epsilon_{2}\epsilon_{2}{\;}^{T},\epsilon_{3}\epsilon_{3}{\;}^{T}$, or the fiber orientation distribution functions (FOD) (Tournier et al., 2004) measured with high-angular resolution diffusion MRI (HARDI) (Tuch et al., 2002) in the brain (Leergaard et al., 2010). Numerous dMRI studies of cortical microstructure in fixed tissues (McNab et al., 2009, 2013; Dyrby et al., 2011; Miller et al., 2011; Kleinnijenhuis et al., 2013; Leuze et al., 2014; Aggarwal et al., 2015; Avram et al., 2022) and *in vivo* (Jaermann et al., 2008; Heidemann et al., 2010; McNab et al., 2013; Kleinnijenhuis et al., 2015; Gulban et al., 2018; Wang et al., 2021), for review see Assaf (2019), suggest that at submillimeter spatial resolution diffusion in the cortex is anisotropic and varies with the cortical folding geometry (McNab et al., 2013; Cottaar et al., 2018; Avram et al., 2022), in good agreement with the cortical cyto- and myeloarchitectonic features observed with histology and other modalities (Nieuwenhuys, 2013). Moreover, HARDI-derived FODs show preferentially radial and tangential components (Kleinnijenhuis et al., 2013; Leuze et al., 2014; Aggarwal et al., 2015) which evoke cortical columns (Petersen, 2007; Yacoub et al., 2008) and layers (Nagy et al., 2013; Bastiani et al., 2016), respectively, that can be observed with post-mortem histological staining. In addition, studies of laminar-specific intra-cortical connectivity measured with diffusion fiber microtractography (Leuze et al., 2014) of cortical FODs (Aggarwal et al., 2015; Gulban et al., 2018) suggest a similar orthogonal (radial and tangential) organization.

Increasing the spatial resolution in dMRI reduces the intravoxel angular dispersion of subvoxel diffusion processes and implicitly the orientational variance of the DTD. At submillimeter spatial resolution, dMRI is sensitive to cortical diffusion anisotropy and allows us to identify the radial and tangential orientations along which diffusion processes align. Recently, a careful survey of the high-resolution dMRI literature (Assaf, 2019) suggests that when different contrast preparations are used to vary the relative contributions of microscopic tissue water pools to net voxel dMRI signal in the cortex, the dominant diffusion orientations, as measured using the DTI eigenvectors or the directions of FOD peaks, remain unaffected even though the relative diffusivities or FOD amplitudes along these orientations may change. At mesoscopic spatial resolutions of a few 100 *μ*m, the orientational characteristics of the dMRI signal remain remarkably consistent across experiments with fixed and live cortical tissues using different T1- and/or T2-weightings, i.e., different echo time (TE), repetition time (TR), or inversion time (TI), diffusion sensitizations (*b*-values) or diffusion/mixing times. These findings imply that at mesoscopic spatial resolutions, subvoxel cortical diffusion tensors from microscopic water pools are coincident along the same dominant (radial and tangential) orientations but may have potentially different diffusion anisotropies and diffusivities. Implicitly, at the mesoscopic length scale, the DTD is predominantly determined by the variations in the shapes (diffusion anisotropies) and sizes (diffusivities) of the microscopic diffusion tensors, rather than by their relative orientations.

In this study, we describe a new framework that simplifies the measurement and analysis of diffusion heterogeneity in microscopic water pools within gray matter using a non-parametric DTD. Specifically, if the voxel size is small enough compared to the radius of curvature of the cortex, we can constrain all the microscopic (subvoxel) diffusion tensors to share the same principal reference frame determined, for instance, by the dyadic of the principal diffusion eigenvectors, ϵ**_1_**, ϵ**_2_**, ϵ**_3_**, measured with DTI. With this constraint, the DTD is completely characterized by the voxel reference frame $\epsilon_{1}\epsilon_{1}{\;}^{T},\epsilon_{2}\epsilon_{2}{\;}^{T},\epsilon_{3}\epsilon_{3}T$, and by the 3D joint distribution of corresponding subvoxel principal diffusivities, λ_1_, λ_2_, λ_3_, which are random variables. This joint probability distribution can be estimated with a 3D Inverse Laplace Transform analysis using only single diffusion encoded (SDE) MR measurements. This practical, non-parametric framework for mapping DTDs, called COnstrained Reference frame diffusion TEnsor Spectroscopic (CORTECS) MRI, has the potential to quantify a wide range of cortical diffusion heterogeneity in healthy or diseased brains.

## 2. Methods

### 2.1. Higher spatial resolution reduces the intravoxel orientational dispersion of dMRI signals

The net diffusion signal in an imaging voxel containing complex tissue microstructure can be described generally using an ensemble of subvoxel (i.e., microscopic) diffusion tensors with different sizes, shapes, and orientations assumed to be in slow exchange, i.e., the diffusion tensor distribution (DTD). Ordinarily, we can quantify DTDs by analyzing diffusion-weighted images (DWIs) acquired with multidimensional diffusion encoding (MDE) (Westin et al., 2016; Topgaard, 2017; Magdoom et al., 2021). The net dMRI voxel signal, *S*, is a function of the tensor-valued encoding variable called the b-tensor, **b**, computed by integrating the time-dependent diffusion gradient waveforms amplitudes, and is related to the underlying DTD, *p*(**D**):


(2) $$\begin{array}{l} S\left(\text{b}\right)=\int_{M_{+}}p\left(\text{D}\right)e^{-\text{b}\cdot \text{D}}d\text{D}, \end{array}$$

where the integral runs over the space or domain of all positive definite matrices, $M_{+}$. Since the random variable D has 6 degrees of freedom, *p*(**D**) is essentially a 6-dimensional joint probability distribution (or correlation spectrum) of the diffusion tensor elements. The high dimensionality and the inherent challenge of defining the subspace of positive-definite random tensor-valued variables, **D**, make solving this problem infeasible in practice, as no closed-form solution exists. Measuring *p*(**D**) requires a prohibitively large number of measurements with a very high signal-to-noise ratio (SNR) and MDE. Previously, approximations to *p*(**D**) have been proposed either by assuming parametric models and/or by using statistical reconstruction algorithms (Jian et al., 2007; Westin et al., 2016; Topgaard, 2017; Magdoom et al., 2021).

**Figure 1:**
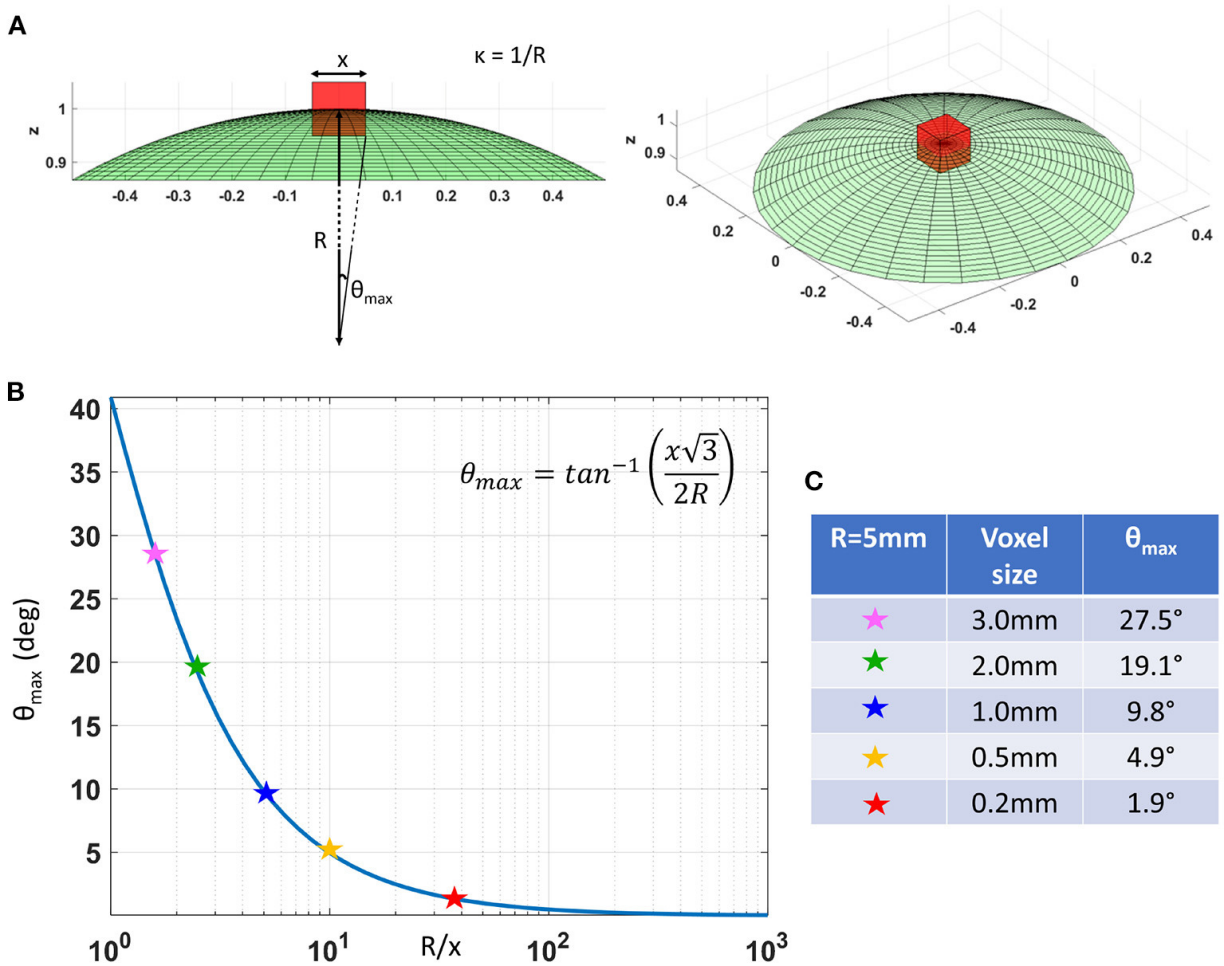
**(A)** As we decrease the voxel size, *x*, relative to the radius of curvature of the tissue (e.g., due to cortical folding), *R*, the intravoxel orientational variance of the continuously varying microstructural reference frame also decreases. For a voxel with an arbitrary orientation relative to the underlying microstructure, the range of intravoxel orientational variation due to tissue curvature is ±θ_*max*_. **(B)** The value of θ_*max*_ decreases rapidly at low spatial resolutions, *R*/*x*, but changes very slowly at higher spatial resolutions, *R*/*x*. **(C)** A quantitative comparison of θ_*max*_ at different voxel sizes assuming a cortical radius of curvature *R* = 5*mm* shows the significant reduction in intravoxel orientational variance due to the effects of anatomical curvature at high spatial resolutions.

In cortical GM the orthogonal coordinate axes along which diffusive fluxes align at the microscopic scale of cellular and subcellular structures (i.e., diffusion length scale) are propagated at larger mesoscopic scales guiding the assembly of these structures into orthogonal tissue architectural patterns of cortical laminae and columns (Nieuwenhuys, 2013; Rubenstein and Rakic, 2020). If the voxel size of dMRI data is significantly smaller than the minimum radius of the curvature of the underlying anatomy (i.e., cortical folding) the orientational variance of subvoxel (microscopic) diffusion processes can be neglected (Figure 1). Microscopic diffusion processes are coincident with the axes of the local microstructural reference frame determined by the cortical cyto- and myeloarchitecture. For a continuously varying cortical anatomy with a minimum radius of curvature, *R*, the range of orientational misalignment between the microscopic diffusion tensors and the voxel reference frame, ±θ_*max*_, in a cubic voxel of side length, *x*, is:


(3) $$\begin{array}{l} \theta_{max}=\tan^{-1}\left(\frac{x\sqrt{3}}{2R}\right) \end{array}$$

**Figure 2:**
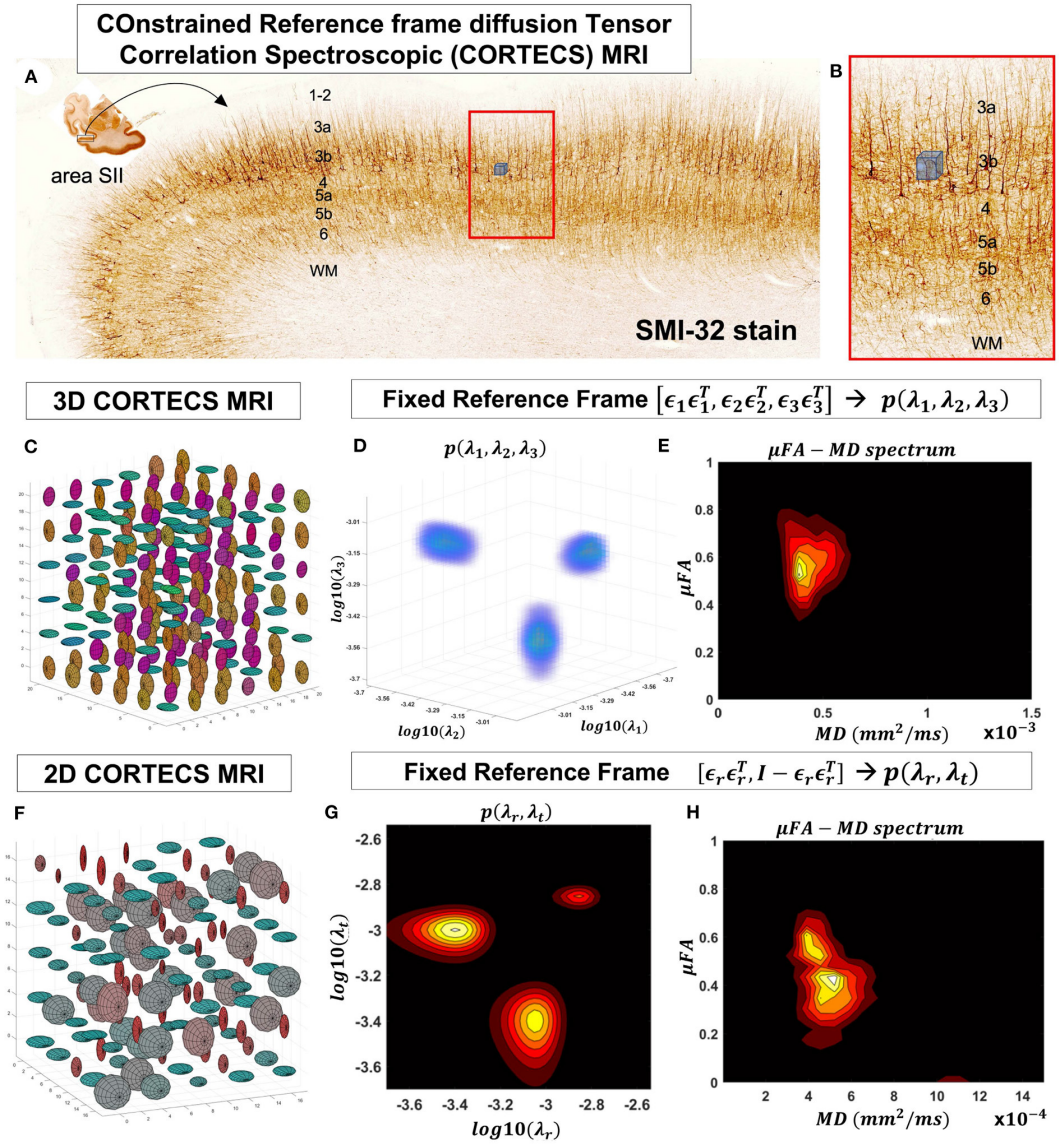
At a mesoscopic length scale cortical cyto- and myeloarchitecture is organized preferentially along the axes of an orthogonal frame of reference **(A)**. If the dMRI spatial resolution is sufficiently small (Figure 1) we can measure DTDs efficiently using the constraints of the CORTECS MRI framework **(B)**. If we constrain all microscopic diffusion tensors to have the same principal axes of diffusion **(C)** we can quantify the DTD as the 3D correlation spectrum of the corresponding principal diffusivities **(D)**. If the microarchitecture varies along a single radial orientation, we can further constrain the DTD to contain only axisymmetric tensors **(F)** and quantify the 2D correlation spectrum of the corresponding radial and tangential diffusivities **(G)**. We can also quantify the shape-size (i.e., microscopic FA-MD) correlation spectra of microscopic tensors from the 3D **(E)** or 2D **(H)** constrained reference frame DTDs (cDTDs).

Figure 1B shows that θ_*max*_ decreases rapidly at low spatial resolutions, $\frac{R}{x}$, but changes slowly at higher values of $\frac{R}{x}$ (Figure 1B). At a spatial resolution of a few hundred micrometers the voxel size is much smaller than the cortical radius of curvature (*R* = 5 mm) leading to very small values of θ_*max*_. Under these circumstances, it is reasonable and practical to constrain all diffusion tensor processes in microscopic water pools throughout the voxel (i.e., the DTD) to be described using the same local orthogonal reference frame (Figures 2A,B).

### 2.2. COnstrained Reference frame diffusion TEnsor Correlation Spectroscopic (CORTECS) MRI

Fixing the local reference frame for all subvoxel tensors has several surprising advantages. First, it significantly reduces the dimensionality of *p*(**D**) and decouples the statistical random variables needed to describe *p*(**D**). Specifically, the 6D vector/tensor random variable, **D**, corresponding to the 6 components (or degrees of freedom) needed to describe the general DTD is reduced to a 3D random variable comprising the three principal diffusivities, λ_1_, λ_2_, λ_3_ along the axes of the fixed voxel frame of reference, $\epsilon_{1}\epsilon_{1}{\;}^{T},\epsilon_{2}\epsilon_{2}{\;}^{T},\epsilon_{3}\epsilon_{3}T$, respectively, which are sufficient to describe the constrained DTDs (cDTDs) within the CORTECS MRI framework (Figures 2C–E). Using the eigenvalue decomposition of the diffusion tensor (Equation 1) we can re-write Equation (2) as a more tractable 3D Inverse Laplace transform (ILT) problem:


$$\begin{array}{l} S\left(\text{b}\right)=\overset{\infty }{\underset{0}{\int }}\overset{\infty }{\underset{0}{\int }}\overset{\infty }{\underset{0}{\int }} \end{array}$$

(4) $$\begin{array}{ll} p(\lambda_{1},\lambda_{2},\lambda_{3})e^{{\;}^{-\lambda_{1}}\epsilon_{1}{\;}^{T}}{\;}^{b\epsilon_{1}-\lambda_{2}\epsilon_{2}{\;}^{T}b\epsilon_{2}-\lambda_{3}\epsilon_{3}{\;}^{T}b\epsilon_{3}}d\lambda_{1}d\lambda_{2}d\lambda_{3}, & \;\;\;\;(4) \end{array}$$

where ${\epsilon_{i}}^{T}\text{b}\epsilon_{i}$ is a non-negative scalar weighting that represents the reciprocal Laplace variable corresponding to λ_*i*_, and **b** is the measurement b-tensor (Westin et al., 2016). We can estimate non-parametric DTDs in the reduced dimensional space of the principal diffusivities by applying the ILT to dMRI data acquired with SDE and/or MDE. Besides the drastic reduction in the computational complexity due to the dimensionality reduction, the CORTECS framework inherently enforces positive definiteness of diffusion tensors by requiring positivity for λ_*i*_.

Another very important advantage of constraining the reference frames of the DTD tensor random variable is that we can measure *p*(λ_1_, λ_2_, λ_3_) using only DWIs acquired with single pulse-field gradient (sPFG), or SDE, a.k.a. linear tensor encoding with rank-1 b-tensors. For a conventional SDE DWI with an arbitrary *b*-value, *b*, and diffusion gradient direction given by the unit vector $\text{g}={\left[g_{x},g_{y},g_{z}\right]}^{T}$, the encoding b-tensor has rank-1, **b** = *b***gg**^*T*^. We can rewrite the signal equation above with respect to the components of **g** expressed in the voxel frame of reference, ${\text{g}}^{\prime }={\left[{g^{\prime }}_{1},{g^{\prime }}_{2},{g^{\prime }}_{3}\right]}^{T}=\left[\epsilon_{1}\epsilon_{2}\epsilon_{3}\right]\text{g}$:


$$\begin{array}{l} S\left(\text{b}\right)=\int_{0}^{\infty }\int_{0}^{\infty }\int_{0}^{\infty } \end{array}$$

(5) $$\begin{array}{l} p\left(\lambda_{1},\lambda_{2},\lambda_{3}\right)e^{-\lambda_{1}b{g^{\prime }}_{1}^{2}}e^{-\lambda_{2}b{g^{\prime }}_{2}^{2}}e^{-\lambda_{3}b{g^{\prime }}_{3}^{2}}d\lambda_{1}d\lambda_{2}d\lambda_{3} \end{array}$$

The factors $b{g^{\prime }}_{i}^{2}$ are the non-negative weighting parameters of the principal diffusivities, λ_*i*_, in the Laplace Transform representation of the signal. We can generate a wide range of joint weighting parameters $b{g^{\prime }}_{i}^{2}$ by varying the *b*-value and diffusion gradient orientations in conventional SDE preparations. Subsequently, from multiple SDE DWIs we can estimate, in each voxel, the correlation spectrum of principal diffusivities, *p*(λ_1_, λ_2_, λ_3_) which quantifies the properties of all microscopic diffusion tensors. Compared to MDE-DWIs, the conventional SDE-DWI can be acquired efficiently using product single pulsed-field gradient (sPFG) spin-echo (SE) diffusion MR sequences (Stejskal and Tanner, 1965) available on all microimaging and clinical MRI scanners. In general, SDE-DWIs can achieve higher *b*-values, shorter echo times (TEs), higher spatial resolution, and/or better SNR than MDE-DWIs using double or triple diffusion encoding (Sjölund et al., 2015). Moreover, the spectral reconstruction of *p*(λ_1_, λ_2_, λ_3_), henceforth referred to as 3D cDTD, does not require statistical methods to enforce positive definiteness but can still benefit from various techniques that may be used to solve ILT-like problems, such as L_2_- or L_1_-norm regularization, compressed sensing (Bai et al., 2015), or constrained optimization (Benjamini et al., 2016), etc.

The microarchitecture of certain tissues can be described more economically and effectively using a single dominant axis. For example, in cortical gray matter, microscopic diffusion processes align with cell and tissue structures (Budde and Annese, 2013; Seehaus et al., 2013, 2015) that are preferentially oriented radially or tangentially with respect to the cortical surface (Kleinnijenhuis et al., 2013; McNab et al., 2013; Leuze et al., 2014). Under these circumstances, we can further simplify the problem and assume that the DTD comprises only tensors with cylindrical symmetry (Figures 2F–H). Thus, the voxel reference frame is determined by a single orientation, $\epsilon_{1}{\epsilon_{1}}^{T}$, i.e., normal to the cortical surface, which implicitly determines the orthogonal, tangential component described by the rank-2 tensor $\epsilon_{2}{\epsilon_{2}}^{T}+\epsilon_{3}{\epsilon_{3}}^{T}=I_{3}-\epsilon_{1}{\epsilon_{1}}^{T}$, where $I_{3}$ is the 3*x*3 identity matrix. We can relate the signal in a voxel with fixed principal axis $\epsilon_{1}{\epsilon_{1}}^{T}$ to a two-dimensional correlation spectrum of principal diffusivities along radial (cortical columns) and tangential (cortical layers) orientations with respect to the cortical surface, *p*(λ_*r*_, λ_*t*_):


(6) $$\begin{array}{l} S\left(\text{b}\right)=\int_{0}^{\infty }\int_{0}^{\infty }p\left(\lambda_{r},\lambda_{t}\right)e^{-\lambda_{r}b\cos^{2}\phi_{\text{g}}}e^{-\lambda_{t}b\sin^{2}\phi_{\text{g}}}d\lambda_{r}d\lambda_{t} \end{array}$$

The parameter $\phi_{\text{g}}=\arccos\left({\epsilon_{1}}^{T}\text{g}\right)$ represents the angle between the applied gradient direction, **g**, and the radial direction of the underlying reference frame, $\epsilon_{1}{\epsilon_{1}}^{T}$, while *p*(λ_*r*_, λ_*t*_) completely determines the corresponding distribution of cylindrically symmetric diffusion tensors, henceforth referred to as the 2D cDTD.

Lastly, in a final simplifying step, if all subvoxel diffusion processes are isotropic, the correlation spectrum of diffusion tensor eigenvalues reduces to a distribution of a single scalar diffusivity random variable, λ_0_, which can be viewed as a 1D cDTD:


(7) $$\begin{array}{l} S\left(\text{b}\right)=\int_{0}^{\infty }p\left(\lambda_{0}\right)e^{-\lambda_{0}b}d\lambda_{0} \end{array}$$

As an aside, we should point out an important connection between 1D cDTD MRI and our previously proposed methods for one- and multidimensional MD spectroscopic MRI using isotropic diffusion encoding (IDE) (Avram et al., 2019, 2021). Mapping non-parametric spectra of MD values in microscopic tissue water pools using multiple IDE measurements does not require that diffusion in these pools is isotropic. Meanwhile, the 1D cDTD MRI spectral reconstruction using Equation (7) correctly quantifies the spectra of water mobilities only if all diffusion processes within the voxel are isotropic, in which case the two methods will provide congruent results.

### 2.3. Mapping distributions and correlation spectra of microscopic fractional anisotropy and mean diffusivity

From the measured cDTD within each voxel, we can compute non-parametric distributions and correlation spectra of any DTI parameters derived from the microscopic diffusion tensors, such as fractional anisotropy (FA) or mean diffusivity (MD). Specifically, we can define a new random variable, α, that quantifies the FA of each microscopic diffusion tensor in the cDTD:


(8) $$\begin{array}{l} \alpha =\sqrt{\frac{1}{2}}\frac{\sqrt{{\left(\lambda_{1}-\lambda_{2}\right)}^{2}+{\left(\lambda_{2}-\lambda_{3}\right)}^{2}+{\left(\lambda_{3}-\lambda_{1}\right)}^{2}}}{\sqrt{\lambda_{1}^{2}+\lambda_{2}^{2}+\lambda_{3}^{2}}} \end{array}$$

From *p*(λ_1_, λ_2_, λ_3_) we can then derive the probability density function (one-dimensional spectrum) of the microscopic tensor FAs, *p*_*FA*_(*α*), which quantifies the cDTD shape heterogeneity non-parametrically. The statistical moments of *p*_*FA*_(α) provide important microstructural parameters, such as the microscopic anisotropy, *μFA*, computed as the mean of *p*_*FA*_(*α*) (Lasic et al., 2014; Westin et al., 2016; Magdoom et al., 2021). Similarly, we can define another cDTD-derived random variable that quantifies the mean diffusivity of each microscopic tensor, *μ* = (λ_1_+λ_2_+λ_3_)/3, and compute its probability density function *p*_*MD*_(*μ*) to describe the spectrum the microscopic tissue water mobilities non-parametrically.

Finally, from *p*(λ_1_, λ_2_, λ_3_) we can also compute non-parametric multidimensional correlation spectra of two or more microscopic DTI metrics. For example, we can quantify non-parametrically the correlations between the shapes and sizes of the diffusion ellipsoids corresponding to the microscopic diffusion tensors by computing the joint probability density function of the two random variables *α* and *μ*, *p*_*FA*−*MD*_(*α*, *μ*). This practical and efficient decomposition of tissue heterogeneity based on diffusion anisotropy and mean diffusivity correlations in microscopic water pools may reveal specific microstructural motifs or patterns potentially relevant to many clinical applications.

### 2.4. A generalization of several multi-tensor diffusion signal models

The CORTECS framework can describe a wide range of heterogeneous diffusion processes in healthy and diseased tissues and subsumes several diffusion tensor signal models. For example, if we constrain $p\left(\lambda_{1},\lambda_{2},\lambda_{3}\right)=\delta (\lambda_{1}-{\lambda^{\prime }}_{1},\lambda_{2}-{\lambda^{\prime }}_{2},\lambda_{3}-{\lambda^{\prime }}_{3}$), 3D cDTD simplifies to conventional DTI with the three mean eigenvalues ${\lambda^{\prime }}_{1},{\lambda^{\prime }}_{2},{\lambda^{\prime }}_{3}$. In this way, 3D cDTD can be viewed as a generalization of high-resolution DTI that quantifies intravoxel diffusion heterogeneity as a non-parametric correlation spectrum of the principal diffusivities in microscopic water pools. To describe multi-exponential or multi-tensor signal decays in heterogeneous tissues (Stanisz et al., 1997; Mulkern et al., 1999; Avram et al., 2020) we can assume that *p*(λ_1_, λ_2_, λ_3_) can be represented as a sum of delta functions (point masses) (Avram et al., 2020). Moreover, the spectroscopic decomposition of the net voxel signal in cDTD makes it easy to disentangle partial volume contributions, such as those from cerebrospinal fluid (CSF), or free water in tissues caused by edema or other processes (Pasternak et al., 2009).

### 2.5. Monte Carlo simulations

We conducted Monte Carlo (MC) simulations to evaluate the numerical stability and accuracy of the voxel-wise estimation of 3D and 2D cDTDs from noisy data. Specifically, starting from ground truth DTDs constrained with fixed voxel reference frames (2D and 3D cDTDs), defined analytically using multidimensional log-normal distributions, respectively, we computed the dMRI signals expected from an experiment using conventional single-diffusion encoded (SDE) DWI measurements with the same gradient orientations and *b*-values as in our fixed-brain experiment described below. Next, from these ground truth signals, we generated 500 instances of noisy measurements by adding Rician noise to simulate real measurements with different SNR levels. From each set of noisy measurements, we computed the corresponding normalized 3D correlation spectra of principal diffusivities, or normalized 2D correlation spectra of radial and tangential diffusivities and compared the statistics of these spectra (mean and standard deviation) to the corresponding ground truth 3D and 2D DTDs, respectively.

### 2.6. Ultra high-resolution dMRI of a fixed macaque monkey brain

The brain of a healthy young adult rhesus macaque monkey (*Macaca mulatta*) weighing 13.55 kg was prepared using a well-controlled perfusion fixation process, as described in Saleem et al. (2021). In brief, the animal was deeply anesthetized with sodium pentobarbital and perfused transcardially with heparinized saline, followed by 4% paraformaldehyde in 0.1 M phosphate buffer (pH 7.4). After perfusion, the brain was removed from the cranium and post-fixed for 8 h in the same buffered paraformaldehyde solution. Following the post-fixation, the brain was transferred into 0.1 M phosphate-buffered saline with sodium azide before the MRI data acquisition. All procedures were carried out under a protocol approved by the Institutional Animal Care and Use Committee of the National Institute of Mental Health (NIMH) and the National Institute of Health (NIH) and adhered to the Guide for the Care and Use of Laboratory Animals (National Research Council).

Based on a preliminary structural MRI scan of the specimen, we fabricated a three-dimensional (3D) brain mold inside a cylindrical acrylic plastic container. The specimen was positioned inside the brain mold which was placed inside a custom 70 mm cylindrical container. The container was filled with Fomblin and gently stirred under a vacuum for 4 h to remove air bubbles. Subsequently, the container was sealed and prepared for MR imaging using a Bruker 7T horizontal-bore MRI scanner and a Bruker 72 mm quadrature RF coil.

We acquired whole-brain diffusion-weighted images (DWIs) with a cubic voxel size of 200*μm*, i.e., a 375 x 320 x 230 imaging matrix on a 7.5 x 6.4 x 4.6 cm field-of-view (FOV), using a diffusion spin-echo 3D echo-planar imaging (EPI) sequence with 50 ms echo time (TE), 650ms repetition time (TR), 18 segments and 1.33 partial Fourier acceleration. We obtained a total of 112 DWIs using multiple *b*-value shells (100, 1,000, 2,500, 4,500, 7,000, and 10,000 *s*/*mm*^2^) with diffusion-encoding gradient orientations (3, 9, 15, 21, 28, and 36, respectively) uniformly sampling the unit sphere on each *b*-value shell and across shells (Koay et al., 2012). The diffusion gradient pulse durations and separations were δ = 6 ms and Δ = 28 ms, respectively. Each DWI volume was acquired using a single average in 52 min. The total duration of the diffusion MRI scan was 93 h and 20 min. We processed all whole-brain high-resolution DWIs with the TORTOISE software pipeline (Pierpaoli et al., 2010) which includes image registration, Gibbs ringing correction (Kellner et al., 2016), denoising (Veraart et al., 2016), corrections for EPI distortion including eddy currents and B0 inhomogeneities using a high-tissue contrast structural magnetization transfer (MT) scan as an anatomical template. To better visualize the preferred diffusion orientations in cortical GM, we computed fiber orientation distribution functions (FODs) and derived direction-encoded color (DEC) images (Dhollander et al., 2015). First, we interpolated the multi-shell dMRI dataset (Özarslan et al., 2013) onto a single densely sampled (128 uniformly sampled orientations) b-shell (*b* = 8, 000*s*/*mm*^2^), then used single-shell single-tissue constrained spherical deconvolution (CSD) in MRtrix (Tournier et al., 2012) to estimate and visualize FODs.

### 2.7. Histological processing

After imaging, the perfusion-fixed brain specimen was prepared for histological processing with five different stains as described in Saleem et al. (2021). In brief, the brain blocks were frozen and serially sectioned through the entire brain at 50*μm* thickness in the coronal plane. Next, five sets of interleaved sections were processed for Parvalbumin (PV), neurofilament protein (SMI-32), choline acetyltransferase (ChAT), cresyl violet (CV), and Acetylcholinesterase (AchE) staining. Finally, we captured high-resolution images of stained sections using a Zeiss high-resolution slide scanner with 5X and 20X objectives. These images were then manually aligned with the corresponding slices from the MRI data for comparison of cortical architectonic features.

### 2.8. 2D CORTECS MRI in the fixed macaque brain

From the distortion-corrected DWIs we estimated fiber orientation distribution functions and compared their orientations in the cortex to those of microscopic structures observed on histological images. We further analyzed the high-resolution DWIs using DTI and estimated the voxel reference frame, $\epsilon_{1}\epsilon_{1}{\;}^{T},\epsilon_{2}\epsilon_{2}{\;}^{T},\epsilon_{3}\epsilon_{3}T$, through eigenvalue decomposition of the net diffusion tensor in each voxel (Equation 1). Subsequently, using the diffusion principal diffusion direction $\epsilon_{1}{\epsilon_{1}}^{T}$, we computed the diffusion weightings of radial and tangential processes, $b\cos^{2}\phi_{\text{g}}$ and $b\sin^{2}\phi_{\text{g}}$, respectively, for each measurement encoding and in each voxel. Finally, we estimated a piecewise continuous approximation of the 2D cDTD correlation spectrum, *p*(λ_*r*_, λ_*t*_), by numerically solving the 2D ILT problem (Equation 6) using linear least-squares error minimization with L2-norm regularization (Hansen, 1992) and positivity constraints, as described in Avram et al. (2019, 2021). The spectral bins of the cDTD reconstruction were defined on a 12 x 12 grid of logarithmically spaced λ_*r*_ and λ_*t*_ values ranging from 0.01 to 2.00 *μm*^2^/*ms*. From the 2D cDTD correlation spectrum *p*(λ_*r*_, λ_*t*_) we derived maps of the marginal distributions of the radial and tangential diffusivities, microscopic FA and MD, as well as the microscopic FA-MD correlation spectra, *p*_*FA*−*MD*_(*α*, *μ*), and related these results to cortical cytoarchitectonic features observed with histology. The microscopic FA-MD correlation spectra were estimated numerically from the cDTDs using an 11 x 11 grid of microscopic FA and MD values. We empirically selected several *ad hoc* spectral domains in the 2D joint distributions *p*(λ_*r*_, λ_*t*_) and *p*_*FA*−*MD*_(*α*, *μ*) to best capture the most prominent spatial-spectral correlations. We compared maps of the signal components corresponding to these domains to the cortical cytoarchitectonic features in the corresponding stained tissue section. The cDTD reconstruction and analysis for the numerical simulations and fixed brain experiments were implemented in MATLAB.

## 3. Results

### 3.1. Monte Carlo simulations

**Figure 3:**
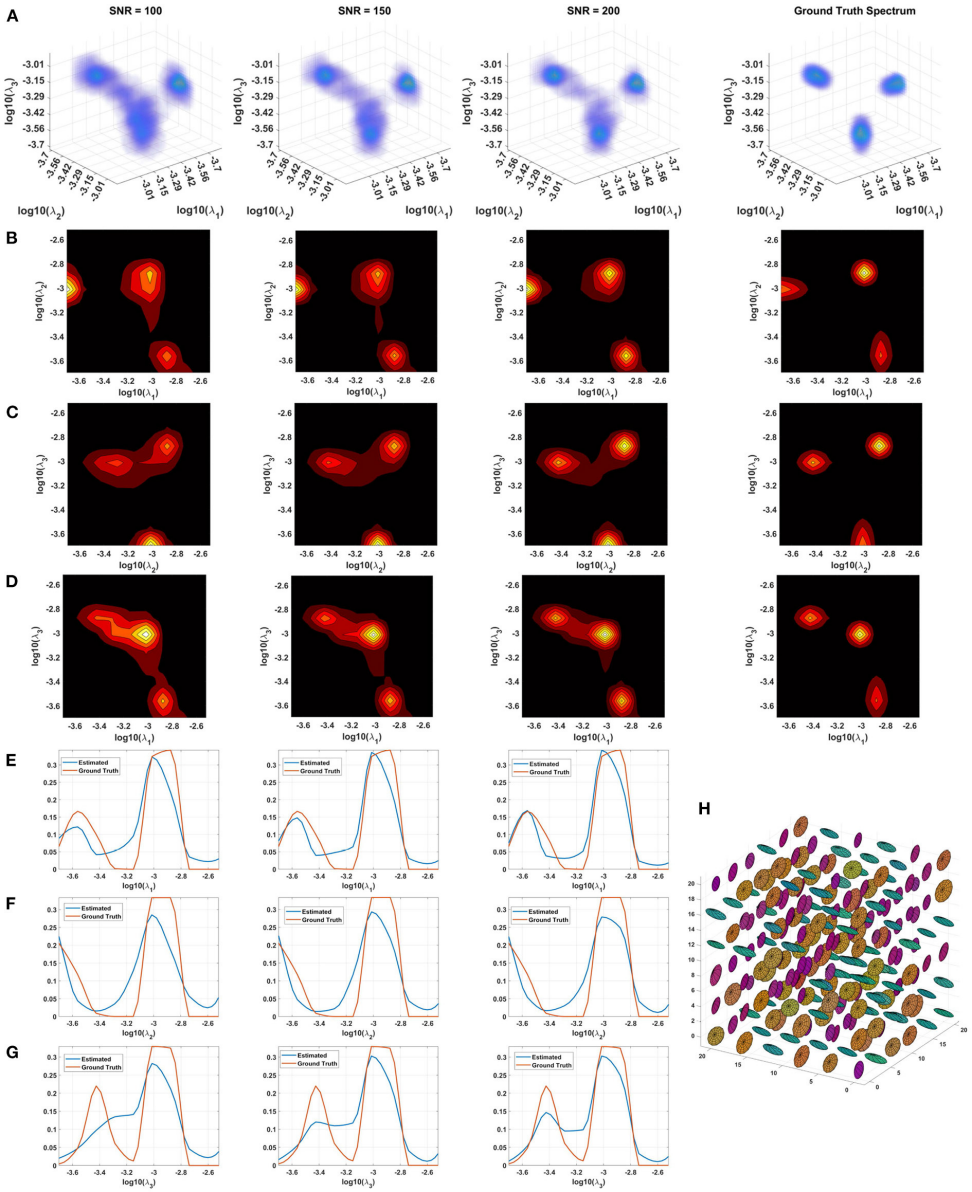
Monte-Carlo simulation results illustrating the accuracy and numerical stability of the 3D cDTD reconstruction as a function of SNR. **(A)**: Log-log-log plots of mean normalized 3D cDTD correlation spectra of the principal diffusivities reconstructed from data with different SNRs. **(B–D)**: Log-log plots of mean normalized 2D marginal distributions derived from the 3D cDTDs in the top row. **(E–G)**: Log plots of the mean normalized 1D marginal distributions derived from the 3D cDTDs in the top row. **(H)**: A numerically simulated illustration of an ensemble of diffusion tensors described by the ground truth 3D cDTD.

**Figure 4:**
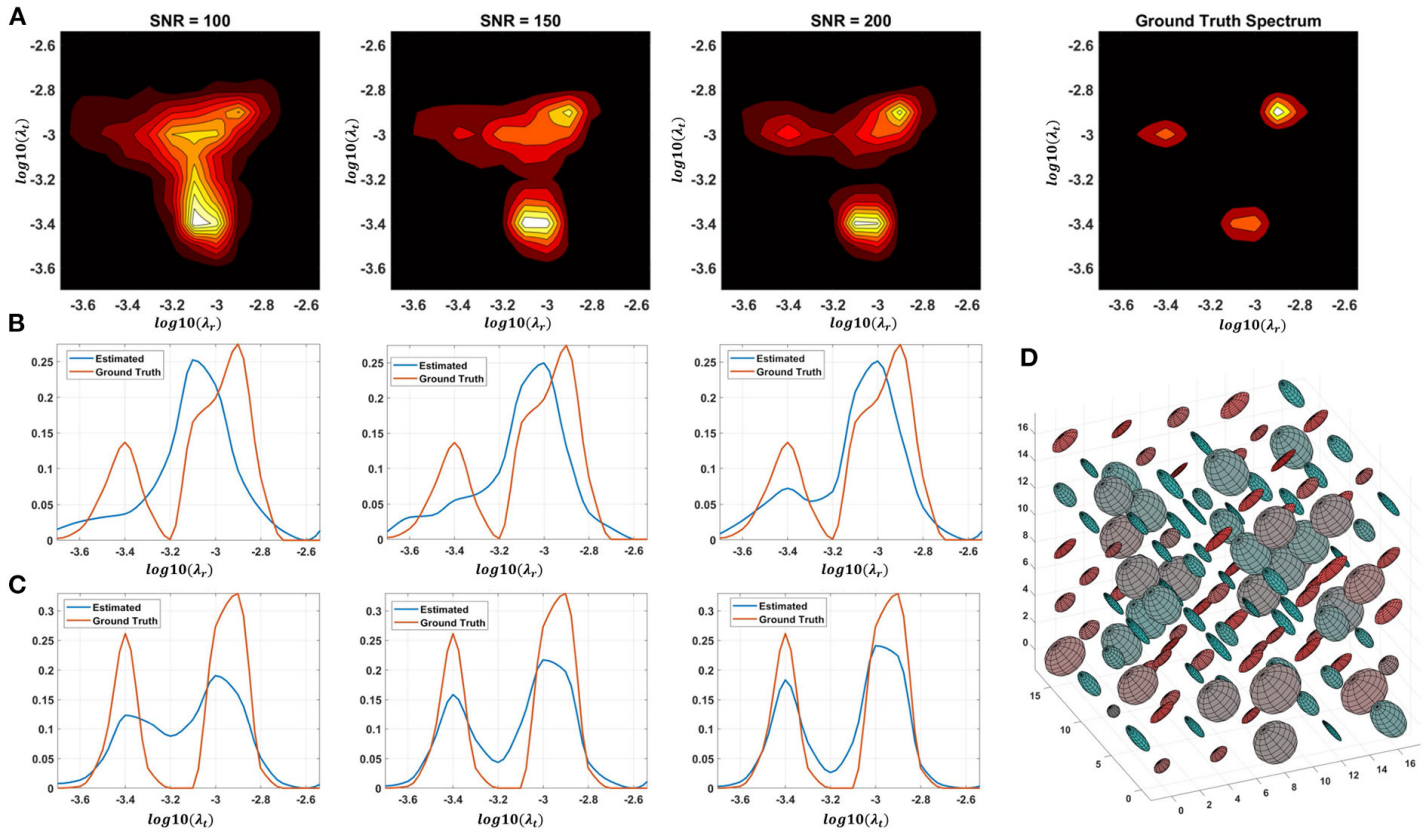
Monte-Carlo simulation results illustrating the accuracy and numerical stability of the 2D cDTD reconstruction as a function of SNR. **(A)**: Log-log plots of mean normalized 2D cDTD correlation spectra of principal diffusivities reconstructed at different SNR levels. **(B,C)**: Log plots of mean normalized 1D marginal distributions derived from the 2D cDTDs in the top row. **(D)**: A numerically simulated illustration of an ensemble of diffusion tensors described by the ground truth 2D cDTD.

Monte Carlo (MC) simulations of 3D and 2D cDTD reconstructions show that it is possible to distinguish subvoxel diffusion tensor processes that are aligned in the same voxel reference frame based on differences in the correlations of their principal diffusivities using experimental designs that contain only SDE measurements and can be achieved with current MRI scanners. Figure 3 shows the MC results for a ground truth 3D cDTD, i.e., correlation spectrum of principal diffusivities, (λ_1_, λ_2_, λ_3_), that consists of a mixture of three multivariate log-normal distributions with peaks at (1.4, 1.0, 0.4), (1.1, 0.2 0.9), and (0.3, 1.3, 1.2) *μm*^2^/*ms* and standard deviations of 0.1 *μm*^2^/*ms*, reflecting the presence of 3 microscopic water pools with distinct diffusion tensor properties. The mean normalized spectra reconstructed from noisy measurements with various SNR levels provide good estimates for the locations and concentrations (i.e., areas under the peaks) of individual signal components. Meanwhile, at higher SNR levels, the exact shapes of the estimated spectral peaks are more accurately resolved. Lower dimensional marginal distributions derived from the 3D cDTDs also reveal the presence of multiple peaks and show improved accuracy at higher SNR levels. Similar results were obtained in MC simulations using 2D cDTDs shown in Figure 4. The ground truth correlation spectrum of radial and tangential diffusivities, *p*(λ_*r*_, λ_*t*_), that defines the 2D cDTD, consists of a mixture of three multivariate log-normal distributions with peaks located at (0.9,0.4), (0.4,1.0), and (1.4,1.4) *μm*^2^/*ms* and standard deviations of 0.1 *μm*^2^/*ms*. The locations and concentrations of these peaks can be estimated over a wide range of SNR levels, with improved accuracy at higher SNRs. Nevertheless, at lower SNR levels the exact shapes of these peaks may be prone to biases. The smoothed appearances of the reconstructed mean normalized spectra in Figures 3, 4 are due to regularization (Hansen, 1992) and, in part, to small variations in the locations and widths of the peaks estimated from data with different instances of noise. Errors in the estimated spectra may be due to measurement noise, the limited number of measurements, and/or the regularization and positivity constraints used to improve the condition number of the spectral reconstruction. To better visualize the SNR-dependence of reconstruction accuracy for the 2D and 3D CORTECS MRI examples shown in Figures 3, 4 we assessed the similarities between the mean normalized spectra reconstructed from data with different SNR levels and the ground truth distributions using the mean-squared error (MSE), structural similarity index (SSIM) (Wang et al., 2004), and the Jensen-Shannon Divergence (JSD) (Lin, 1991) (please see Supplementary Figure S1).

The spectral resolution depends on the number of measurements with different encodings, the SNR level, and the use of constraints and regularization for spectral reconstruction. For a fixed SNR and a wide range of signal weightings (e.g., *b*-values), slowly decaying components have a better contrast-to-noise ratio (CNR) than fast decaying ones and can therefore be resolved with higher spectral resolution. The resulting non-uniform spectral resolution is not unique to CORTECS MRI but is inherent to the data required by all multidimensional relaxation and diffusion spectroscopic MRI methods. These techniques aim to disentangle multiexponential processes by quantifying the underlying distribution of decay constants non-parametrically using an ILT-like reconstruction from a finite set of measurements. The spectral resolution could be improved by using more advanced spectral reconstruction algorithms that rely on statistical methods (Prange and Song, 2009), compressed sensing (Bai et al., 2015), various constraints (Benjamini and Basser, 2016), Bayesian estimation (McGivney et al., 2018), or deep learning (Pirk et al., 2020).

In general, the presence of the fixative and the reduced temperature (room temperature vs. body temperature) decreases the diffusivities in fixed tissues compared to those observed in the live human brain (Dyrby et al., 2011). It is important to note that if we scale all diffusivities by any factor, say 3, and the *b*-values used in our experiment by its inverse, i.e., 1/3, all signal attenuations, *e*^−*bD*^, remain unchanged. Consequently, the Monte Carlo simulations with different SNR levels obtained using fixed brain diffusivities and this study's experimental design with $b_{max}=10,000s/mm^{2}$ also accurately describe an experiment in which all tissue diffusivities are scaled by a factor of 3 simulating *in vivo* conditions and all *b*-values are scaled by a factor of 1/3, i.e., $b_{max}=3,333s/mm^{2}$, simulating clinical scan parameters.

### 3.2. Comparison of dMRI and histological sections

**Figure 5:**
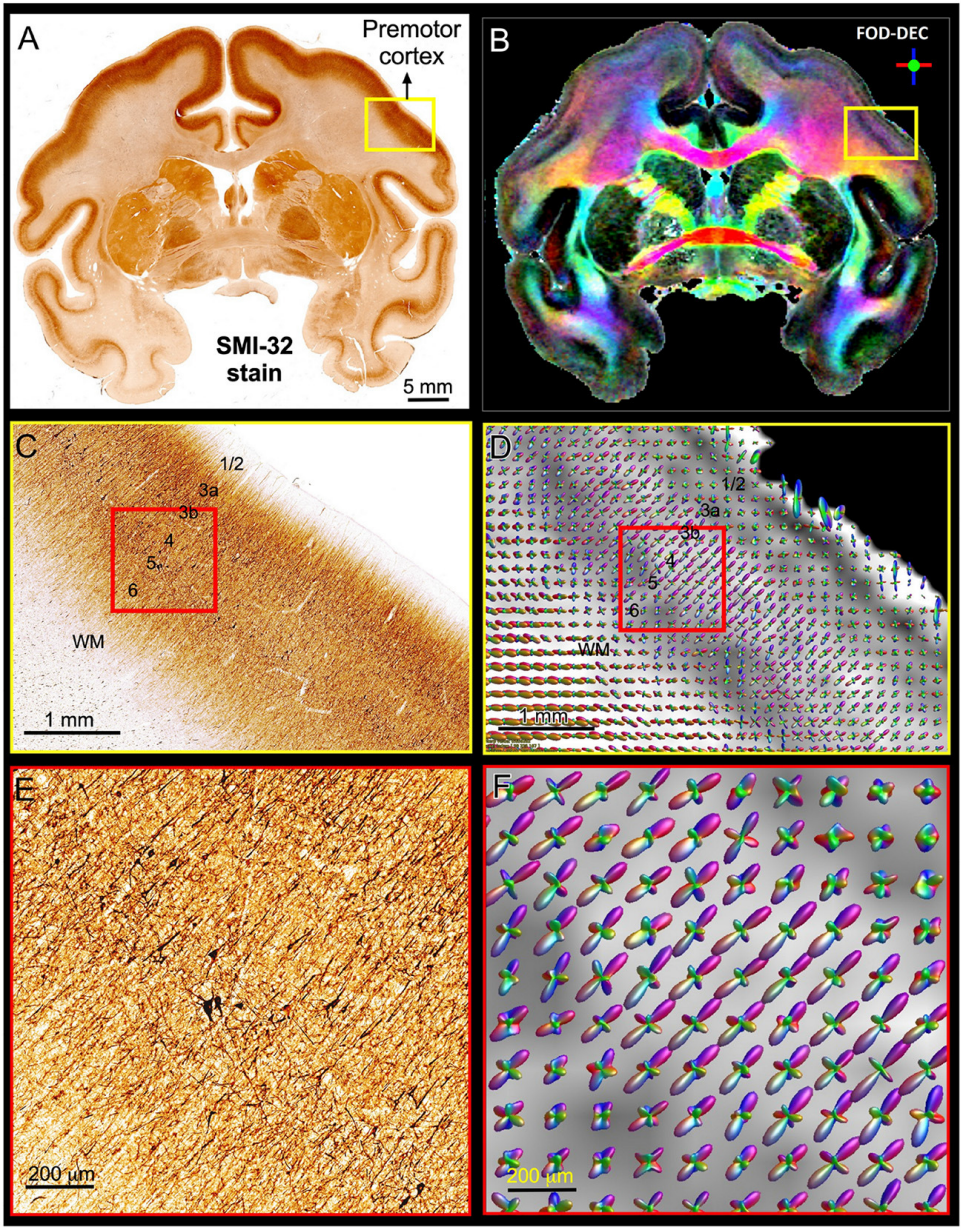
Views of the brain anatomy at the macroscopic scale in a coronal tissue section stained with SMI-32 **(A)** and the FOD-DEC image in a matched MRI slice **(B)** showing the dependence of the principal diffusion direction on the cortical folding geometry. **(C,D)**: Enlarged views of the mesoscopic scale of the histological image and FOD glyphs corresponding to the yellow outlines in **(A,B)**, respectively. The cortical architecture shows a laminar pattern of radially coherent cell processes with different densities (labeled cortical layers). **(E,F)**: Enlarged views of the histological image and FOD glyphs corresponding to the red outline in **(C,D)**. The locally coherent alignment of FOD peaks **(F)** matches the microstructural tissue architecture comprising radial and tangential cell processes **(E)**.

Figure 5 shows a multi-scale side-by-side comparison of a coronal section stained with SMI-32 and the corresponding dMRI data in a representative region of the dorsal premotor cortex. At the macroscopic scale (Figures 5A,B) we can clearly see that the dominant diffusion direction in the FOD-DEC image (Dhollander et al., 2015) (Figure 5B) varies continuously along the cortical ribbon and remains perpendicular to the cortical surface. At the mesoscopic scale (Figures 5C,D) the curvature of the cortex becomes less prominent and the tissue architecture reveals radially oriented neurofilaments in pyramidal neurons with a staining intensity that varies in a laminar pattern reflecting distinct cortical layers. The FODs measured with dMRI in the same region (Figure 5C) show a good alignment of water diffusion with the dominant orientation of the local microstructure at the scale of hundreds of micrometers. A careful visual inspection of the SMI-32 section at high magnification (Figure 5E) reveals the presence of cell processes oriented radially and tangentially with respect to the cortical surface. The contribution of tangential processes contributes to the slight differences in SMI-32 staining intensities across cortical layers. At this scale, the grid-like cortical architecture is clearly observable in the orthogonal orientations of the FOD peaks which vary continuously and coherently across multiple voxels (Figure 5F). These observations confirm similar results from numerous high-resolution dMRI studies and suggest that cortical diffusion processes are locally oriented along orthogonal reference frames that match the tissue architecture and do not change significantly at the scale of a few hundred micrometers, providing a strong justification for describing diffusion processes at smaller length scales with the same fixed locally orthogonal reference frame.

### 3.3. Cortical architectonic features revealed with CORTECS MRI

The SNR was estimated as the non-diffusion attenuated magnitude signal averaged in a region-of-interest (ROI) divided by the noise standard deviation measured in several ROIs outside the brain (Afzali et al., 2021) using the raw magnitude signals (before post-processing). The cortical SNR varied between 50 and 120. Several imaging artifacts may contribute to an underestimation of the SNR, including:

1. Ghosting/aliasing artifacts induced by the vibration of gradient coils (potentially leading to noise overestimation),
2. Inaccurate calibration of the transmit and receive gains causing a non-zero background in the reconstructed images (potentially leading to noise overestimation), and
3. Spatial inhomogeneities in the B1 sensitivity (potentially leading to tissue signal underestimation).

**Figure 6:**
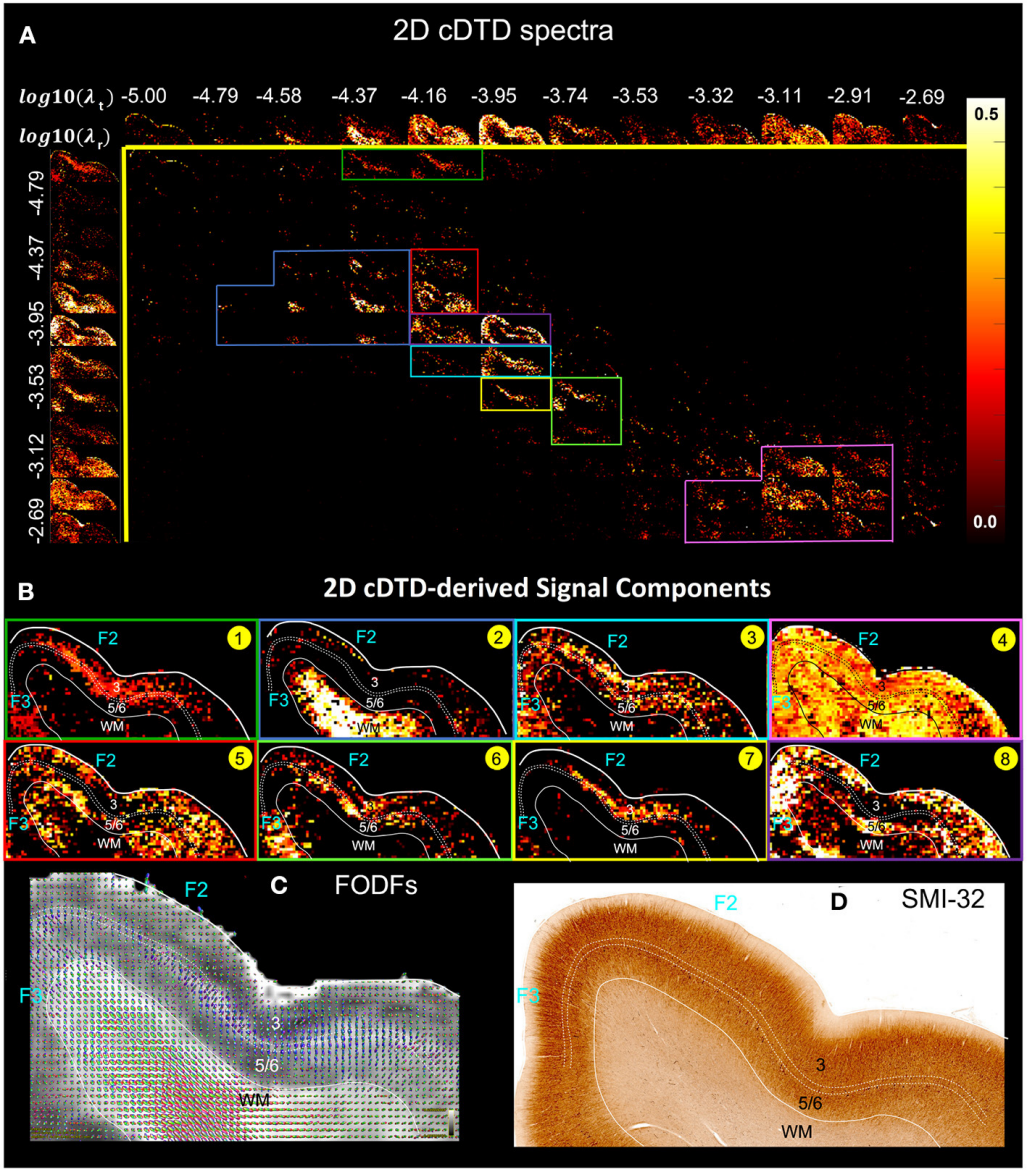
**(A)** Spectral component maps of normalized 2D correlation spectra of radial and tangential diffusivities in a section of the cortex from Figure 5A. Top row: Spectral component maps of the normalized 1D marginal distribution of tangential diffusivity, λ_*t*_; Left column: Spectral component maps of the normalized 1D marginal distribution of radial diffusivity, λ_*r*_. **(B)** Tissue component maps derived by integrating the 2D cDTD spectral components over empirically defined spectral regions of interest delineated with different colors show good specificity to cortical layers. **(C)** Corresponding FODs. **(D)** Corresponding SMI-32 stained section.

Our preliminary results of imaging 2D cDTDs in cortical GM reveal diffusion processes with distinct joint radial and tangential diffusivities and different specificities across cortical domains and layers. In Figure 6, the spectral component images on the diagonal line λ_*r*_ = λ_*t*_ represent isotropic diffusion processes, while those below and above this line quantify anisotropic processes that can be described using prolate and oblate diffusion tensors, respectively. Comparing the maps of the 1D marginal distributions of λ_*r*_ (Figure 6, left column) and λ_*t*_ (Figure 6, top row) we found that the spectra of radial diffusivities in tissue microenvironments provides slightly better sensitivity to cortical layers than those of tangential diffusivities. Figure 6B quantitatively maps the concentrations of eight distinct microscopic diffusion processes which were computed by integrating the 2D cDTDs over spectral domains (Figure 6A, color-coded outlines) defined empirically based on spatial correlations of spectral components. The resulting signal component maps show high specificity to various cortical layers and were in good agreement with the diffusion orientational features observed in the FOD maps (Figure 6C). For example, high concentrations of radial microscopic diffusion processes were observed primarily in the mid-cortical layers (Figure 6B, Components 1 and 7) and in subcortical WM (Figure 6B, Component 2), while high concentrations of more isotropic and tangential microscopic diffusion processes were observed primarily in the superficial and deep cortical layers (Figure 6B, Components 5 and 8). The spatial distribution of Component 3 (Figure 6B) in layer 3 and part of layers 5 and 6 matched with the distribution of non-pyramidal neurons in the parvalbumin stained section (not shown in Figure 6). Meanwhile, the dense and patchy distribution of Component 6 (Figure 6B) localized mainly in layer 5 corresponded to the intensely stained pyramidal neurons in this layer in AchE- (not shown in Figure 6) and SMI-32-stained sections (Figure 6D).

### 3.4. Shape-size correlation spectra derived from the cDTD distributions

**Figure 7:**
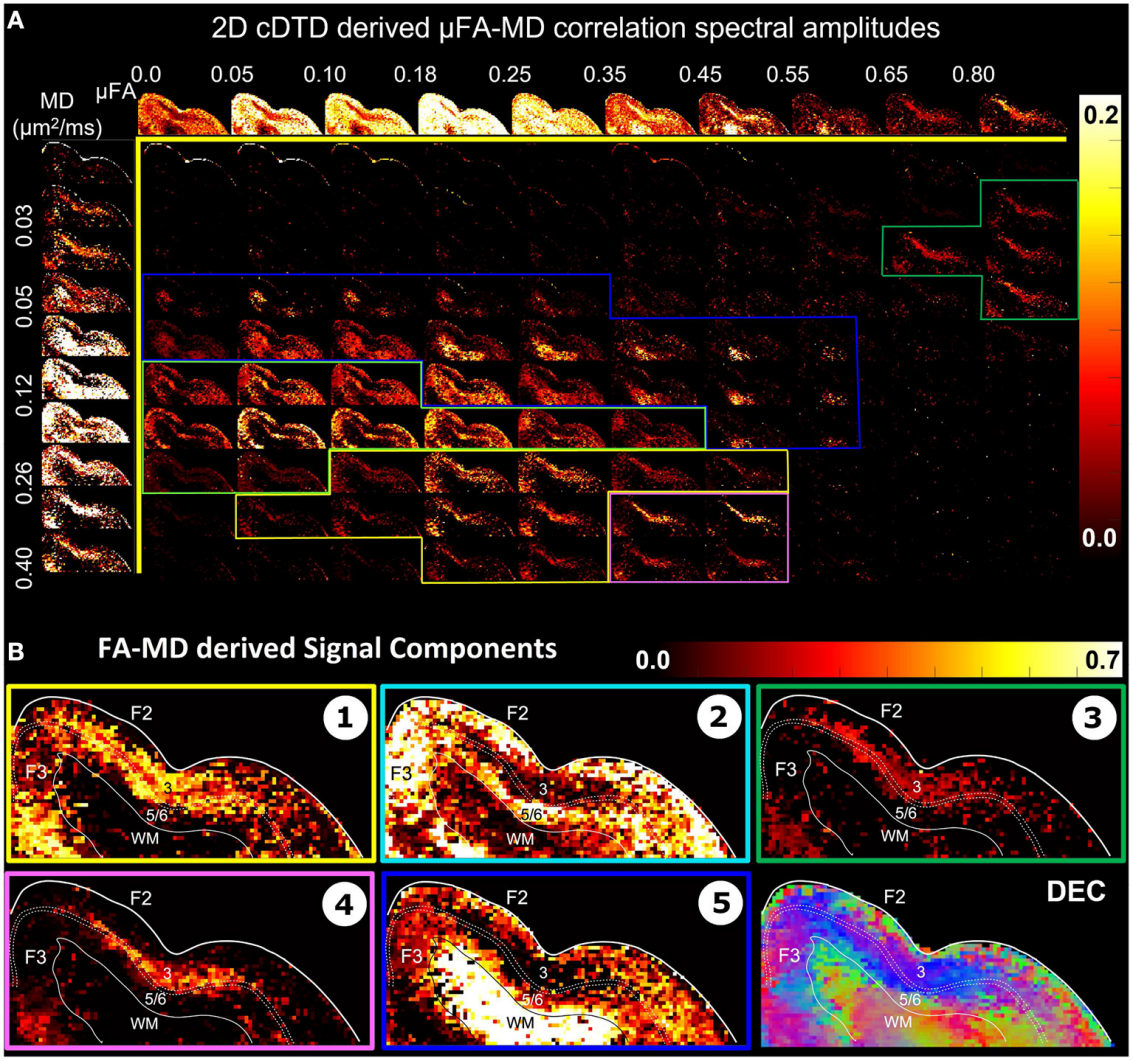
**(A)** Spectral amplitude maps of normalized 2D *μFA*−*MD* correlation spectra in the section of the cortex from Figure 6. Top row: Spectral component maps of the normalized 1D marginal distribution of microscopic fractional anisotropy, *μFA*; Left column: Spectral component maps of the normalized 1D marginal distribution of the microscopic diffusion tensor mean diffusivities. **(B)** Tissue component maps derived by integrating the 2D *μFA* − *MD* distributions over empirically defined spectral regions reveal strong contrast in the mid-cortical areas.

The distributions *p*_*FA*_(*α*) (Figure 7A, top row) and *p*_*MD*_(*μ*) (Figure 7A, left column) are derived from the non-parametric 2D cDTDs in Figure 6 and quantify the shapes (FAs) and sizes (MDs), respectively, of the microscopic diffusion tensors. Moments of these distributions may provide important information about the underlying microstructural heterogeneity. The first moment of *p*_*FA*_(*α*) quantifies the *μFA* (Lasic et al., 2014) quantifies the average anisotropy of the microscopic diffusion tensors, while the second moment (variance) quantifies the shape heterogeneity of these tensors (please see Supplementary Figure S2).

The 2D *μFA*−*MD* correlation spectral amplitude maps in Figure 7 provide a tally of the joint shape-size characteristics of the microscopic diffusion tensors of the DTD as a new means to characterize tissue microstructure. The largest concentrations of isotropic microscopic diffusion processes (*μFA* < 0.18) were observed in the upper cortical layers, and to a lesser extent, in layer 5. The most anisotropic diffusion processes (*μFA* > 0.35) were localized in the mid-cortical layers and in the subcortical white matter. The signal in subcortical WM voxels spanned a large range of *μFA* values, potentially reflecting diffusion processes with a larger intravoxel orientational variance (e.g., bending/crossing WM fibers) that may be inadequately described by the cDTDs. The 1D marginal distributions of both the microscopic fractional anisotropies (Figure 7A, top row) and mean diffusivities (Figure 7A, left column) derived from the *μFA*−*MD* spectra show layer-specific motifs that allow us to distinguish between superficial, mid, and deep cortical layers. Spectra of MD values in microscopic water pools show the highest concentration of low MD processes in WM (Figure 7A, component 3), and a mixture of diffusion processes with low and high water mobilities in the mid-cortical layers, potentially indicating important differences in cellularity between these layers. Meanwhile, spectra of *μFA* values revealed predominantly anisotropic diffusion processes in the mid-cortical layers and more isotropic diffusion processes in the superficial and deep layers. Figure 7B quantifies the spatial distributions and concentrations of five distinct microscopic diffusion components obtained by integrating the 2D *μFA*−*MD* correlation spectra over empirically defined spectral domains (Figure 7B, color-coded outlines). In Figure 7B, Components 1, 3, and 4 are specific to the midcortical layers, while Components 2 and 5 are localized almost exclusively in the superficial/deep cortical layers and in subcortical WM, respectively. Component 3 in the *μFA*−*MD* maps (Figure 7B), shows very high *μFA* and likely corresponds in part to the signal from Component 1 in the λ_*r*_−λ_*t*_ maps (Figure 6B) with a small λ_*r*_ and large λ_*t*_. It appears to suggest the presence of a small concentration of highly anisotropic oblate microscopic diffusion tensors.

It is likely that this component reflects restricted water diffusion within tangentially oriented tissue and cell processes (e.g., neurites, neurofilaments) which are powder-averaged within the plane of the mid-cortical layers (Figure 5E). In this case, the restricted tangential diffusion processes cannot be accurately modeled using tensors (e.g., a powder-average of prolate tensors) and the tangential diffusivities derived with DTD MRI, in general, do not accurately reflect the water diffusivities in different pools (e.g., inside or outside the dendrites). Nevertheless, even if the cDTD-derived diffusivity and anisotropies spectral components may not be quantitative (i.e., biased), they could still provide important clinical information about the density of tangentially oriented neurites or the transverse tortuosity of the extracellular space.

### 3.5. Potential sources of errors

The accuracy of the measured cDTD spectra depends on several experimental factors such as the number of measurements, the diffusion gradient directions, *b*-values, as well as the SNR. During the voxel-wise cDTD reconstruction, the dMRI signals are decomposed along the axes of the local frame of reference. Consequently, for the same diffusion encoding (i.e., same DWI) the effective diffusion weightings (Equation 6) of the radial and tangential diffusivities, $b\cos^{2}\phi_{\text{g}}$ and $b\sin^{2}\phi_{\text{g}}$, respectively, may differ from voxel to voxel. To prevent biases due to the orientations of the local microstructure in the reconstructed cDTD maps it is important that the diffusion encodings uniformly sample the unit sphere for each *b*-value and across *b*-values. Moreover, one could augment the CORTECS MRI experimental design by adding measurements with MDE.

Two additional potential sources of errors in the spatial-spectral mapping of microscopic diffusion processes with CORTECS MRI in this study may arise from 1. inaccuracies in estimating the DTI-derived reference frame, and 2. inconsistencies between the axes of the DTI-derived reference frames across neighboring voxels due to the sorting bias of the diffusion tensor eigenvalue decomposition (Pierpaoli and Basser, 1996). Both sources of errors become more prominent when the dMRI voxel signal is more isotropic. If the signal is isotropic in 3D, the principal diffusion axes are poorly-defined and the estimated diffusion reference frames may be inconsistent across adjacent voxels.

In cortical tissues, the DTI and, more generally, the dMRI signals show little anisotropy within the plane tangent to the cortical surface, even at high spatial resolutions and high *b*-values. As a result, it is difficult to uniquely define orthogonal principal diffusion axes within the tangential orientation. Instead, we can use a more economical characterization of the microscopic diffusion processes using a distribution of axisymmetric tensors. The resulting 2D cDTDs are determined by *p*(λ_*r*_, λ_*t*_) and the dominant (radial) diffusion direction normal to the cortical surface, which can be reliably estimated in the cortex. The DEC map in Figure 5 shows a continuously varying radial diffusion orientation along the cortical ribbon. Despite variations in diffusion anisotropy across cortical layers the principal axis of diffusion corresponding to the largest DTI eigenvalue, $\epsilon_{1}{\epsilon_{1}}^{T}$, can be reliably estimated throughout the cortex and is consistently oriented normal to the cortical surface. Moreover, this orientation matches that of the largest FOD peak in each corresponding voxel. The side peaks of the FODs are consistently oriented in the tangential plane perpendicular to the radial direction, supporting the orthogonal alignment of diffusion processes, in good agreement with findings from previous high-resolution cortical dMRI studies (Kleinnijenhuis et al., 2013; Leuze et al., 2014; Aggarwal et al., 2015).

However, more generally, when DTI data is acquired with lower spatial resolution, low FA values in the cortex can bias the measurement of the radial direction that determines the 2D cDTD reference frame in each voxel. In this situation, it may be possible to use higher *b*-values (or longer diffusion times) to improve the sensitivity to the orientational features of the dMRI signal, and/or to estimate the voxel reference frame more reliably from the directions of the largest FOD peaks. Alternatively, one could derive a cortical reference frame from the curvature of the cortex measured using a structural scan with good GM-WM contrast as a proxy for the diffusion reference frame (Avram et al., 2020) or use spline interpolation of the diffusion tensor field (Pajevic et al., 2002) in low FA voxels, to derive a continuously varying reference frame that is consistent throughout the cortex.

## 4. Discussion

The CORTECS framework greatly simplifies the data acquisition and spectral reconstruction requirements for high-resolution DTD MRI and subsumes several previously proposed multi-tensor diffusion models. It provides a practical and feasible approach to non-parametric quantitation of microstructural heterogeneity in healthy and diseased tissues. At its core, the framework relies on the observation that, in tissues with a consistent well-defined architecture, such as the cortex, as we increase the spatial resolution from the scale of a conventional dMRI voxel (≈ 2*mm*) to the mesoscopic scale much smaller than the radius of cortical curvature, the intravoxel angular dispersion of diffusion processes decreases rapidly. At the mesoscopic scale of a few hundred micrometers diffusion processes in distinct tissue microenvironments, e.g., associated with myelin, intra-, extra-axonal water, remain largely coincident along the axes of a common reference frame determined by the local tissue architecture. At this length scale, the intravoxel angular dispersion due to cortical folding is significantly reduced and differences between subvoxel (microscopic) diffusion processes are primarily characterized by their principal diffusivities. Correlations between principal diffusivities explain most of the microscopic diffusion heterogeneity. They determine the anisotropies and mean diffusivities of the microscopic diffusion tensors, i.e., the shapes and sizes of their diffusion ellipsoids, rather than their relative orientations, allowing us to constrain the DTD reconstruction.

### 4.1. The persistence of the principal diffusion orientations for various signal weightings

The basis of constraining cortical diffusion processes to be oriented along local orthogonal directions in neural tissue has many lines of support. Direct observations of cortical cyto- and myelo-architectonic features with optical and 3D electron microscopy reveal dominant radial and tangential orientations. Meanwhile, histological validation studies using high spatial and angular resolution dMRI with a range of mesoscopic spatial resolutions have repeatedly shown that in neural tissues the preferential diffusion directions align with the dominant orientation of the underlying microstructure. Moreover, results from numerous high-resolution dMRI studies suggest that when the relative signal contributions (weightings) from specific water pools are altered using different signal preparations the principal axes of the diffusion tensors and the orientations of the dominant FOD peaks in the voxel do not change (Assaf, 2019). Concretely, the dominant diffusion orientations do not change significantly in experiments with a wide range of echo times (T2-weightings) (Avram, 2011; Avram et al., 2012), repetition times, inversion times (T1-weightings) (Assaf, 2019), *b*-values (diffusivity weightings) and diffusion times (chemical exchange and restriction weightings). Furthermore, *in vivo* experiments combining diffusion MRI and magnetization transfer (MT) preparation indicate that in white matter fibers the principal diffusion directions of myelin water and non-myelin water pools are coincident (Avram et al., 2010). Similarly, *in vivo* diffusion tensor spectroscopy experiments of neuronal-specific metabolites, such as NAA have shown that diffusion processes in intra- and extracellular water pools are also aligned with the diffusion reference frame of the voxel (Ronen et al., 2013). The persistence of the reference frame under various signal preparations suggests that the intravoxel orientational heterogeneity is dominated by the curvature of the macroscopic anatomy (e.g., cortical folding, fanning/bending WM pathways), and that water diffusion in specific microenvironments of neural tissues can be described adequately with a singular reference frame defined by the mesoscopic architecture. Finally, constraining subvoxel cortical diffusion tensor processes to the local reference frame of the mesoscopic voxel may also be justified with arguments from developmental biology.

### 4.2. Orthogonal reference frames in neurodevelopment

During morphogenesis, diffusion-reaction processes can establish orthogonal concentration gradients (Turing, 1952; Gregor et al., 2005) to support the efficient transport of macromolecules such as growth and inhibitory factors. It is believed that in early embryogenesis this mechanism (Gregor et al., 2005; Lefévre and Mangin, 2010) leads to the formation of the principal axes of embryonic development: rostro-caudal, medio-lateral, and dorso-ventral (Kingsbury, 1920). Similarly, during early brain development diffusion-reaction processes at the microscopic scale, e.g., ≈ 10 − 50 *μm*, likely guide the growth of elongated cellular and sub-cellular structures, such as neurofilaments, axons and dendrites, which in turn, provide a scaffold for the diffusive migration and active transport of macromolecules over longer distances. The progressive elaboration of the orthogonal reference frame provides a plausible explanation for the architecture of cortical columns, laminae, and capillaries, at the mesoscopic scales of ≈ 100 − 500*μm*. Diffusion MRI studies in the late stages of fetal neurodevelopment and newborns have shown a decrease in the radial coherence of diffusion processes (McKinstry et al., 2002; Vasung et al., 2010; Takahashi et al., 2011; Dudink et al., 2015; Khan et al., 2019).

More generally, several theories of brain development (Van Essen, 1997; Lefévre and Mangin, 2010; Wedeen et al., 2012; Chen et al., 2013) suggest to different extents, that similar locally orthogonal reference frames may be observed in WM at high spatial resolution. The intravoxel angular dispersion in WM voxels depends on the curvature of the fiber pathways (e.g., due to bending and fanning) as well as the presence of fiber crossings. The radii of curvature due to bending (e.g., corpus callosum) or fanning (e.g., corticospinal tract) in WM pathways are typically larger than those of the cortical folding geometry (e.g., sulci and gyri), even for short-range U-fibers. Consequently, at the mesoscopic spatial resolutions required for CORTECS MRI, the residual intravoxel orientational variation of diffusion processes in WM is due primarily to the crossing angles of subvoxel fiber populations. CORTECS MRI may be applicable in regions containing a single homogeneous WM pathway (i.e., no crossings), such as the corpus callosum, but not in most WM voxels that contain fiber populations that do not cross at orthogonal orientations. Nevertheless, the framework could provide an independent method to test the hypothesized local orthogonality (Tax et al., 2016, 2017) at various spatial resolutions.

### 4.3. The dimensionality reduction of cDTDs

Current approaches for imaging DTDs and/or their features require SDE and MDE measurements and include parametric models using SDE (Jian et al., 2007) and combinations of SDE and MDE measurements (Lasic et al., 2014; Szczepankiewicz et al., 2016; Westin et al., 2016; Henriques et al., 2020; Magdoom et al., 2021) as well as non-parametric methods (Topgaard, 2017). Parametric DTD models approximate the solution using analytical functions such as a Wishart distribution (Jian et al., 2007) or a constrained normal tensor-variate distribution (Magdoom et al., 2021). While such analytical approximations can estimate DTDs from fewer measurements and lower SNR levels, they drastically limit the space of admissible DTDs to those described by a handful of degrees of freedom (i.e., parameters or coefficients). The reconstructed DTDs may provide biased assessments in voxels affected by partial volume contributions from tissues with very different diffusion properties and may not accurately capture the range of unknown tissue alterations that occur in disease. Non-parametric or spectroscopic DTD reconstruction methods (Topgaard, 2017) can describe an arbitrary range of tissue compositions but, due to the large spectral dimensionality of the problem, require many MDE DWIs with high SNR and computationally intensive statistical reconstruction methods to enforce positive definiteness of the solution.

For a general, unconstrained non-parametric DTD, the microscopic diffusion tensors can have arbitrary orientations (Equation 2). Consequently, the 6-dimensional random variable of the DTD must support both positive and negative off-diagonal tensor elements and cannot be analyzed with conventional ILT methods. To overcome this limitation, the DTD reconstruction requires computationally intensive statistical methods (Topgaard, 2017; Magdoom et al., 2021) to enforce positive definiteness constraints that ensure the physicality of the microscopic diffusion tensors. Alternatively, if we describe the DTD using the principal diffusivities, λ_1_, λ_2_, λ_3_ and the three Euler angles *ϕ, ψ, θ*, which define the orientations of the orthonormal directions ϵ_1_, ϵ_2_, ϵ_3_ in Equation (1), then *ϕ, ψ, θ* create a trigonometric dependence in the signal equation. The key insight of the CORTECS MRI framework is that in tissues with well-defined, orthogonal architectures, sampling the spatial dimensions more densely, i.e., increasing the spatial resolution, reduces the intravoxel angular dispersion. This allows us to restrict the 3 degrees of freedom that determine the orientations of the tensor random variable, i.e. the three Euler angles, and thus reduce the domain of the DTD to the orthogonal non-negative 3D space of principal diffusivity random variables that guarantees positive definiteness and can be solved with a conventional ILT reconstruction techniques. This trade-off between spatial resolution and spectral dimensionality has several important implications for the clinical translation of non-parametric DTD MRI.

### 4.4. Data acquisition requirements for CORTECS MRI

In general, the SNR requirements for multidimensional spectral (i.e., non-parametric) reconstruction algorithms scale exponentially with the dimensionality of the problem. For a 2D spectral reconstruction, an SNR of 100 allows us to measure signal attenuated concurrently by a factor of 10 along two independent spectral dimensions. Meanwhile, to achieve the same effective dynamic range per dimension for a 4D spectral reconstruction, we need an SNR of 10,000. While such nominal SNR levels may be achievable on clinical scanners by using sufficiently large voxel sizes, the integrity of the data acquired *in vivo* may be corrupted (Avram et al., 2019, 2021) by:

1. Imaging artifacts such as ghosting/aliasing, eddy current induced distortions, or Gibbs ringing, which typically represent ≈ 1 − 2% of the tissue signal; and
2. Partial volume inconsistencies across DWIs due to subject and physiological motion (e.g., blood flow, pulsations, etc.).

In routine clinical MRI scans, e.g., T1W, T2W, DTI, typical SNR levels are between 20 and 50, and these signal artifacts on the order of ≈2% are barely visible. However, for an *in vivo* SNR = 10,000, these signal instabilities produce an artifact-to-noise ratio of ≈200, potentially biasing the estimation of non-parametric DTDs in high dimensional spaces (e.g., 4D or 6D) and rendering them unsuitable for clinical translation.

On the other hand, CORTECS MRI measures 3D or 2D correlation spectra using efficient diffusion preparations (SDEs), fewer measurement encodings (data points), and SNR levels that may be achieved for ultra-high resolution *in vivo* dMRI in the near future. Advances in various technologies including the design of high-field MRI scanners (Feinberg et al., 2021), high-performance gradient coils (Foo et al., 2020; Feinberg et al., 2021; Huang et al., 2021), high-density RF coil arrays (Keil et al., 2013; Hendriks et al., 2019), as well as efficient high-resolution dMRI pulse sequences (Feinberg et al., 2010; Avram et al., 2014a; Setsompop et al., 2018), image acquisition and reconstruction strategies (Feinberg et al., 2010; Setsompop et al., 2018), and experimental protocols (Avram et al., 2018, 2019; Nilsson et al., 2020) can be integrated synergistically in state-of-the-art MRI systems (Foo et al., 2020; Feinberg et al., 2021; Huang et al., 2021) to achieve the spatial resolution, scanning efficiency, and diffusion sensitizations required for *in vivo* CORTECS MRI.

In our experiment, the acquisition of each high-resolution DWI volume required 52 min. This relatively long duration scan duration is due to the use of:

1. A large imaging matrix of 375 x 320 x 230 needed for whole-brain coverage at 200 *μm* resolution, and
2. 3D diffusion spin echo EPI sequence with segmented k-space acquisition and a relatively long TR of 650 ms.

The TR was chosen so as to minimize gradient heating (i.e., limit the gradient duty cycle), and included a 150ms duration for excitation, diffusion preparation, and EPI readout, and a 500 ms idle duration. For clinical imaging, both factors can be significantly reduced. Firstly, using a multi-slice spin-echo diffusion EPI sequence with multiband capabilities one could acquire each DWI volume efficiently (negligible idle duration) in a single TR of 5–10 s, albeit at a lower SNR. Secondly, it is important to point out that the requirement for high spatial resolution in CORTECS MRI does not necessarily imply a prohibitively long scan duration. Unlike dMRI fiber tractography, CORTECS dMRI does not require whole-brain data. Using outer-volume suppression, reduced FOV, or ZOOM EPI one could significantly reduce the imaging matrix size and scan duration while still maintaining the required spatial resolution for *in vivo* scans with human subjects. On the other hand, the scan duration requirement of conventional non-parametric DTD methods is inherently limited by the very large number of encodings needed to sample the high-dimensional space exhaustively, even when scanning with a reduced FOV.

### 4.5. Spatial resolution requirements for CORTECS MRI

The major drawback of CORTECS MRI compared to conventional (unconstrained) nonparametric DTD methods is the prerequisite of sufficiently high spatial resolution. The spatial resolution at which we can adopt a common reference frame for all subvoxel diffusion tensors depends on the cortical folding geometry and may vary across the brain. A useful quantity to characterize the validity of this assumption is the dimensionless ratio between the voxel size, *x*, and the minimum radius of curvature of the macroscopic anatomy (e.g., cortical folding, or bending/fanning of WM fibers), *R*. If this ratio is sufficiently small, we can ignore the orientational variations of subvoxel diffusion processes. For example, as shown in Figure 1, for a minimum radius of curvature *R* = 5*mm* in the macaque monkey cortex and a voxel size of *x* = 0.2*mm* used in this study, the expected maximum intravoxel angular deviation between the microstructural and voxel reference frames due to the continuously varying cortical folding geometry, *θ*_*max*_, given by Equation (3) is ±1.9°. This angular deviation is smaller than even the most ambitious estimates of the angular resolution limits in diffusion MRI fiber tractography and is unlikely to bias the estimated cDTD spectra. HARDI experiments using well-calibrated diffusion phantoms with overlapping, highly anisotropic coherent structures oriented at different angles cannot typically resolve diffusion processes due to fibers crossing at angles < 10°, even when a large number of gradient orientations with large *b*-values and high SNR levels are used in microimaging or clinical scanners (Perrin et al., 2005; Guise et al., 2016).

More generally, we can use the analytical relation between *x*/*R* and θ_*max*_ in Equation (3) as a reference for estimating the spatial resolution requirements for 2D CORTECS. To achieve a $\theta_{max}<10^{•}$ comparable to the known angular resolution limits of dMRI we need a value of *x*/*R* = 0.2 (Figure 1). The maximum radius of curvature in the adult human brain is 4 mm (Van Essen and Drury, 1997). Consequently, a voxel size smaller than 0.8 mm may be sufficient for whole-brain 2D CORTECS in the human brain (Wang et al., 2021). Future clinical feasibility studies will explore the sensitivity of the reconstruction to the residual intravoxel orientational dispersion and the possibility of achieving the desired resolution in human scans using reduced field-of-view imaging on state-of-the-art MRI scanners (Huang et al., 2021).

The high spatial resolution requirement in CORTECS MRI can lead to significantly longer acquisition time per volume (i.e., per diffusion encoding), when compared to conventional (unconstrained) nonparametric DTD MRI methods. These methods require large imaging voxel volumes to achieve the very high SNR and signal dynamic range needed for 6D or 4D DTD reconstructions and can be affected by signal artifacts. Moreover, these methods also require a large number of joint (multidimensional) encodings to comprehensively sample the high-dimensional parameter space, thereby offsetting potential savings in the total scan duration that may be gained by imaging a smaller matrix size (i.e., larger voxels), when compared to CORTECS MRI. Most importantly, however, the 6D DTDs measured in voxels of ≈ 3*mm* do not provide any information about the relative spatial distribution of subvoxel diffusion tensors, i.e., at length scales smaller than ≈ 3*mm*. Due to its high spatial resolution requirement, CORTECS MRI explicitly measures the relative spatial distributions (and relative orientations!) of diffusion tensor processes at much finer length scales, e.g., down to 200 *μm* in our study, providing significantly more information. Compared to conventional DTD methods, this higher spatial resolution in CORTECS MRI may provide a more accurate localization and improved sensitivity in the detection of subtle pathological tissue changes, for example in the early stages of neurodegeneration.

### 4.6. Potential for quantifying diffusion time dependence

All DTD MRI methods assume that the voxel can be viewed as an ensemble of non-exchanging Gaussian (i.e., freely diffusing) subvoxel water pools within which the diffusive motions of spins are described with tensors whose corresponding ellipsoids have different sizes, shapes, and orientations. In biological tissues, cellular and subcellular structures can present microscopic restrictions and hindrances producing a time-dependent (non-Gaussian) diffusion in certain water pools. To address this limitation, the MDE-based DTD frameworks (Topgaard, 2017), can be extended to include diffusion time dependence (Lundell et al., 2019), and/or analyzed using parametric models (Henriques et al., 2020). The characteristics of time-dependent DTDs can yield important tissue microstructural information about the distribution of compartment shapes and sizes (Lundell et al., 2019; Henriques et al., 2020) that classical MDE experiments sought to measure (Koch and Finsterbusch, 2008; Avram et al., 2013b; Benjamini et al., 2016; Komlosh et al., 2018). However, it can be troublesome to incorporate the dependence of diffusion processes on the time-varying diffusion gradient waveforms into the signal equation, even for MDE preparations with well-defined diffusion time parameters such as those using double pulsed field gradients (Mitra, 1995; Avram et al., 2013b), or rotating field gradients (Avram et al., 2014b). Conversely, the diffusion time dependence of SDE measurements can provide similar information to MDE measurements (Jespersen, 2012) and is described by a well-defined parameter Δ, the separation between the start times of the two diffusion gradient pulses. Moreover, since the voxel reference frame does not change significantly with diffusion time (Assaf, 2019), we can directly extend the CORTECS framework to map time-dependent cDTDs by repeating the experiment with multiple diffusion times. Imaging correlation spectra of diffusion-time-dependent principal diffusivities in microscopic water pools may provide important pathophysiological information about microscopic restrictions, chemical exchange, and water transport (Nilsson et al., 2013).

### 4.7. Relation to other dMRI methods

The non-parametric cDTD signal representation can be viewed as a multi-tensor generalization of high-resolution DTI. It subsumes several dMRI signal representations and parametric tissue diffusion models, including bi-exponential decay models (Stanisz et al., 1997), free-water elimination (Pasternak et al., 2009), multicompartment tissue models (Stanisz et al., 1997), among others (Panagiotaki et al., 2012), and enables their cross-validation and harmonization. Features of cDTDs measured with CORTECS MRI can inform the design of more efficient dMRI experiments using SDEs and MDEs to measure parametric DTDs and tensor mixture models for specific biological and clinical applications. Moreover, it provides an independent method for deriving DTD-related quantities, such as the non-parametric distribution of subvoxel MD values which can be measured efficiently in a 6 min clinical scan (Avram et al., 2019). In this way, the proposed framework may help test the validity of various DTD methods and guide their development toward achieving higher spatial resolution and greater biological specificity.

The ability to quantify tissue properties non-parametrically is crucial to our understanding of disease progression, tissue regeneration, and neurodevelopment. By quantifying subvoxel DTDs non-parametrically we can identify the most prominent spectral features such as the shapes and peaks or multimodal clusters associated with specific pathophysiological changes. Once we learn these spectral signatures, we can model the CORTECS-derived 2D or 3D cDTDs using analytical functions determined by only a few parameters. Disease-specific parametric cDTD could be reconstructed swiftly and efficiently from data acquired with lower SNR and a smaller number of encodings.

### 4.8. Further improvements in biological specificity

The correlation spectrum of principal diffusivities may reveal signal contributions from specific tissue components, such as intra-axonal, extracellular, or myelin water whose diffusion tensors may be coincident and are therefore difficult to disentangle based on orientational diffusion characteristics such as FODs derived from HARDI data. A further improvement in biological specificity may be achieved by integrating the cDTD measurements with multidimensional relaxation MRI methods (Benjamini and Basser, 2017; Kim et al., 2017) which measure the net voxel signal as a superposition of contributions from subvoxel water pools with different joint T1-, T2- and diffusion properties. However, with the addition of new dimensions for contrast encoding, most implementations of diffusion-relaxation correlation MRI on clinical scanners require larger datasets, higher SNR levels as well as the use of sophisticated pulse sequences and algorithms to reconstruct five-dimensional (Reymbaut et al., 2021) or six-dimensional (de Almeida Martins et al., 2021) correlation spectra. We have recently proposed a more practical two-dimensional diffusion-relaxation MRI method for efficiently mapping T1-MD correlation spectra using isotropic diffusion encoded (IDE) DWIs (Avram et al., 2021). Similarly, the CORTECS framework adds the minimum number of dimensions (principal diffusivities) needed to efficiently combine T1- or T2- relaxation with diffusion tensor spectroscopic imaging.

### 4.9. Potential applications to neuroscience and neuroradiology

Mapping water pools in specific cortical microenvironments based on their diffusion tensor properties quantitatively and efficiently could have numerous applications in neuroradiology and neuroscience. It may improve the diagnosis of neurodevelopmental disorders and allow us to specifically disentangle contributions from increased dendritic arborization and reductions in radial glial fibers to the cortical microstructural changes observed in newborns. In addition, it may provide biomarkers for early detection of cortical microstructural changes occurring in epilepsy (Lampinen et al., 2020), cancer (Szczepankiewicz et al., 2016), traumatic brain injury (Komlosh et al., 2018), stroke (Alves et al., 2022), or multiple sclerosis (He et al., 2021). Mapping correlations between cortical diffusion processes with CORTECS MRI could quantify specific cellular/tissue components providing new parameters for automatic cortical parcellation and layer segmentation algorithms. Relating these layer-specific components to input and output signaling in cortical areas could allow us to study intracortical connectivity and gain valuable insight into the directionality of information flows (signaling) in functional networks throughout the connectome (Olman et al., 2012; Ugurbil et al., 2013). Because it requires only SDE data, CORTECS MRI can be applied retrospectively to analyze existing high-resolution diffusion MRI data sets. Finally, while this study focuses on quantifying diffusion in cortical gray matter, CORTECS MRI may also be applicable to other organized tissues with varying degrees of macroscopic and microscopic diffusion anisotropies such as in white matter, kidney medulla, heart muscle, skeletal muscle, ligaments, tendons, etc.

## 5. Conclusions

This study provides a new framework for empirical and biologically specific analyzes of subvoxel diffusion heterogeneity in brain tissue using conventional high-resolution dMRI. From the non-parametric cDTDs we can derive additional spectral and scalar parameters, such as the joint size-shape distribution of microscopic diffusion tensors. Our preliminary results in the macaque monkey cortex reveal diffusion components that correlate well with distinct architectonic features. Although currently feasible mainly for fixed tissue experiments, CORTECS MRI has the potential to advance the clinical translation of DTD MRI and its optimization for specific applications in clinical and basic sciences. Features of cDTD spectra may help better delineate cortical layers and areas in healthy subjects and may provide new biomarkers for finding subtle cortical abnormalities underlying focal dysplasia in epilepsy, microbleeds in traumatic brain injury, metastatic cancers, etc.

## Data availability statement

The raw data supporting the conclusions of this article will be made available by the authors, without undue reservation.

## Ethics statement

The animal study was reviewed and approved by the Institutional Animal Care and Use Committee of the National Institute of Mental Health (NIMH) and the National Institute of Health (NIH).

## Author contributions

AA: conceptualization, methodology, software, implementation, investigation, data acquisition, data curation, writing–original draft, writing–review and editing, visualization, supervision, and project administration. KS: histological tissue processing and writing–review and editing. PB: conceptualization, writing–review and editing, and funding acquisition. All authors contributed to the article and approved the submitted version.

## References

[B1] Afzali M. Pieciak T. Newman S. Garyfallidis E. Özarslan E. Cheng H.. (2021). The sensitivity of diffusion mri to microstructural properties and experimental factors. J. Neurosci. Methods 347, 108951. 10.1016/j.jneumeth.2020.108951 33017644 PMC7762827

[B2] Aggarwal M. Nauen D. W. Troncoso J. C. Mori S. (2015). Probing region-specific microstructure of human cortical areas using high angular and spatial resolution diffusion MRI. Neuroimage 105, 198–207. 10.1016/j.neuroimage.2014.10.053 25449747 PMC4262592

[B3] Alves R. Henriques R. N. Kerkel L. Chavarras C. Jespersen S. N. Shemesh N. (2022). Correlation Tensor MRI deciphers underlying kurtosis sources in stroke. Neuroimage 247, 118833. 10.1016/j.neuroimage.2021.118833 34929382

[B4] Amunts K. Zilles K. (2015). Architectonic mapping of the human brain beyond Brodmann. Neuron 88, 1086–1107. 10.1016/j.neuron.2015.12.001 26687219

[B5] Assaf Y. (2019). Imaging laminar structures in the gray matter with diffusion MRI. Neuroimage 197, 677–688. 10.1016/j.neuroimage.2017.12.096 29309898

[B6] Assaf Y. Basser P. J. (2005). Composite hindered and restricted model of diffusion (CHARMED) MR imaging of the human brain. Neuroimage 27, 48–58. 10.1016/j.neuroimage.2005.03.042 15979342

[B7] Avram A. V. (2011). Diffusion Tensor Imaging of Myelin Water (Thesis). Duke University.

[B8] Avram A. V. Guidon A. Liu C. Song A. W. (2012). Multi-TE diffusion kurtosis imaging *in vivo*, in Proceedings of nternational Society for Magnetic Resonance in Medicine, Vol. 20 (Melbourne), 3270.

[B9] Avram A. V. Guidon A. Song A. W. (2010). Myelin water weighted diffusion tensor imaging. Neuroimage 53, 132–138. 10.1016/j.neuroimage.2010.06.019 20587369

[B10] Avram A. V. Guidon A. Truong T.-K. Liu C. Song A. W. (2014a). Dynamic and inherent B0 correction for DTI using stimulated echo spiral imaging. Magn. Reson. Med. 71, 1044–1053. 10.1002/mrm.24767 23630029 PMC3760972

[B11] Avram A. V. Özarslan E. Sarlls J. E. Basser P. J. (2013b). In vivo detection of microscopic anisotropy using quadruple pulsed-field gradient (qPFG) diffusion MRI on a clinical scanner. Neuroimage 64, 229–239. 10.1016/j.neuroimage.2012.08.048 22939872 PMC3520437

[B12] Avram A. V. Saleem K. S. Komlosh M. E. Yen C. C. Ye F. Q. Basser P. J. (2022). High-resolution cortical MAP-MRI reveals areal borders and laminar substructures observed with histological staining. Neuroimage 264, 119653. 10.1016/j.neuroimage.2022.119653 36257490

[B13] Avram A. V. Saleem K. S. Ye F. Q. Yen C. C. Komlosh M. E. Basser P. J. (2020). “Modeling cortical architectonic features by analyzing diffusion mri data in the cortical reference frame,” in Proceedings of the 28th Annual Meeting of the ISMRM, Vol. 28, 713.

[B14] Avram A. V. Sarlls J. E. Barnett A. S. Özarslan E. Thomas C. Irfanoglu M. O.. (2016). Clinical feasibility of using mean apparent propagator (MAP) MRI to characterize brain tissue microstructure. Neuroimage 127, 422–434. 10.1016/j.neuroimage.2015.11.027 26584864 PMC4755846

[B15] Avram A. V. Sarlls J. E. Basser P. J. (2013a). Whole-brain assessment of microscopic anisotropy using multiple pulse-field gradient (mPFG) diffusion MRI, in Proceedings of the 22nd Annual Meeting of the ISMRM, Vol. 21 (Slat Lake City, Utah), 2072.

[B16] Avram A. V. Sarlls J. E. Basser P. J. (2019). Measuring non-parametric distributions of intravoxel mean diffusivities using a clinical MRI scanner. Neuroimage 185, 255–262. 10.1016/j.neuroimage.2018.10.030 30326294 PMC6289178

[B17] Avram A. V. Sarlls J. E. Basser P. J. (2021). Whole-brain imaging of Subvoxel T1-diffusion correlation spectra in human subjects. Front. Neurosci. 15, 671465. 10.3389/fnins.2021.671465 34177451 PMC8232058

[B18] Avram A. V. Sarlls J. E. Basser P. J. Özarslan E. (2014b). Rotating field gradient (RFG) diffusion MRI for mapping 3D orientation distribution functions (ODFs) in the human brain, in Proceedings of the 22nd Annual Meeting of the ISMRM, Vol. 22 (Milan), 4453.

[B19] Avram A. V. Sarlls J. E. Hutchinson E. Basser P. J. (2018). Efficient experimental designs for isotropic generalized diffusion tensor MRI (IGDTI). Magn. Reson. Med. 79, 180–194. 10.1002/mrm.26656 28480613 PMC5675833

[B20] Bai R. Cloninger A. Czaja W. Basser P. J. (2015). Efficient 2D MRI relaxometry using compressed sensing. J. Magn. Reson. 255, 88–99. 10.1016/j.jmr.2015.04.002 25917134

[B21] Basser P. J. Mattiello J. LeBihan D. (1994a). Estimation of the effective self-diffusion tensor from the NMR spin echo. J. Magn. Reson. B 103, 247–254. 10.1006/jmrb.1994.1037 8019776

[B22] Basser P. J. Mattiello J. LeBihan D. (1994b). MR diffusion tensor spectroscopy and imaging. Biophys. J. 66, 259–267. 10.1016/S0006-3495(94)80775-1 8130344 PMC1275686

[B23] Bastiani M. Oros-Peusquens A. M. Seehaus A. Brenner D. Mllenhoff K. Celik A.. (2016). Automatic segmentation of human cortical layer-complexes and architectural areas using Ex vivo diffusion MRI and its validation. Front. Neurosci. 10, 487. 10.3389/fnins.2016.00487 27891069 PMC5102896

[B24] Benjamini D. Basser P. J. (2016). Use of marginal distributions constrained optimization (MADCO) for accelerated 2D MRI relaxometry and diffusometry. J. Magn. Reson. 271, 40–45. 10.1016/j.jmr.2016.08.004 27543810 PMC5026962

[B25] Benjamini D. Basser P. J. (2017). Magnetic resonance microdynamic imaging reveals distinct tissue microenvironments. Neuroimage 163, 183–196. 10.1016/j.neuroimage.2017.09.033 28943412 PMC5716914

[B26] Benjamini D. Komlosh M. E. Holtzclaw L. A. Nevo U. Basser P. J. (2016). White matter microstructure from nonparametric axon diameter distribution mapping. Neuroimage 135, 333–344. 10.1016/j.neuroimage.2016.04.052 27126002 PMC4916903

[B27] Brodmann K. (1909). Vergleichende Lokalisationslehre der Grosshirnrinde in ihren Prinzipien dargestellt auf Grund des Zellenbaues. (Leipzig: Barth).

[B28] Budde M. D. Annese J. (2013). Quantification of anisotropy and fiber orientation in human brain histological sections. Front. Integr. Neurosci. 7, 3. 10.3389/fnint.2013.00003 23378830 PMC3561729

[B29] Cajal R. Y. (1909). Histologie du Systme Nerveux de l'homme et des vertébrés. Maloine, Paris.

[B30] Callaghan P. Komlosh M. (2002). Locally anisotropic motion in a macroscopically isotropic system: displacement correlations measured using double pulsed gradient spin-echo NMR. Magn. Reson. Chem. 40, S15–S19. 10.1002/mrc.1122

[B31] Chen H. Zhang T. Guo L. Li K. Yu X. Li L.. (2013). Coevolution of gyral folding and structural connection patterns in primate brains. Cereb. Cortex 23, 1208–1217. 10.1093/cercor/bhs113 22586139 PMC3615352

[B32] Cory D. Garroway A. Miller J. (1990). Applications of spin transport as a probe of local geometry, in Abstracts of Papers of the American Chemical Society, Vol. 199 (Washington, DC: American Chemical Society), 105-POLY.

[B33] Cottaar M. Bastiani M. Chen C. Dikranian K. Van Essen D. Behrens T. E.. (2018). A gyral coordinate system predictive of fibre orientations. Neuroimage 176, 417–430. 10.1016/j.neuroimage.2018.04.040 29684644 PMC6319607

[B34] de Almeida Martins J. P. Tax C. M. W. Reymbaut A. Szczepankiewicz F. Chamberland M. Jones D. K.. (2021). Computing and visualising intra-voxel orientation-specific relaxation–diffusion features in the human brain. Hum. Brain Mapp. 42, 310–328. 10.1002/hbm.25224 33022844 PMC7776010

[B35] Dhollander T. Smith R. E. Tournier J.-D. Jeurissen B. Connelly A. (2015). Time to move on: an fod-based dec map to replace dti's trademark DEC FA, in Conference: 23rd International Society of Magnetic Resonance in Medicine, Vol. 23 (Toronto), 1027.

[B36] Dudink J. Pieterman K. Leemans A. Kleinnijenhuis M. van Cappellen van Walsum A. Hoebeek F. (2015). Recent advancements in diffusion MRI for investigating cortical development after preterm birth–potential and pitfalls. Front. Hum. Neurosci. 8, 1066. 10.3389/fnhum.2014.01066 25653607 PMC4301014

[B37] Dyrby T. B. Baaré W. F. Alexander D. C. Jelsing J. Garde E. Søgaard L. V. (2011). An ex vivo imaging pipeline for producing high-quality and high-resolution diffusion-weighted imaging datasets. Hum. Brain Mapp. 32, 544–563. 10.1002/hbm.21043 20945352 PMC6870191

[B38] Feinberg D. Dietz P. Liu C. Setsompop K. Mukherjee P. Wald L. L.. (2021). Design and development of a next-generation 7T human brain scanner with high-performance gradient coil and dense RF arrays, in Proceedings of the 29th Annual Meeting of the ISMRM, Vol. 29, 562.

[B39] Feinberg D. A. Moeller S. Smith S. M. Auerbach E. Ramanna S. Glasser M. F.. (2010). Multiplexed echo planar imaging for sub-second whole brain FMRI and fast diffusion imaging. PLoS ONE 5, e15710. 10.1371/journal.pone.0015710 21187930 PMC3004955

[B40] Foo T. K. F. Tan E. T. Vermilyea M. E. Hua Y. Fiveland E. W. Piel J. E.. (2020). Highly efficient head-only magnetic field insert gradient coil for achieving simultaneous high gradient amplitude and slew rate at 3.0T (MAGNUS) for brain microstructure imaging. Magn. Reson. Med. 83, 2356–2369. 10.1002/mrm.28087 31763726

[B41] Gregor T. Bialek W. van Steveninck R. R. D. R Tank D. W. Wieschaus E. F. (2005). Diffusion and scaling during early embryonic pattern formation. Proc. Natl. Acad. Sci. U.S.A. 102, 18403–18407. 10.1073/pnas.0509483102 16352710 PMC1311912

[B42] Guise C. Fernandes M. M. Nøbrega J. M. Pathak S. Schneider W. Fangueiro R. (2016). Hollow polypropylene yarns as a biomimetic brain phantom for the validation of high-definition fiber tractography imaging. ACS Appl. Mater. Interfaces 8, 29960–29967. 10.1021/acsami.6b09809 27723307

[B43] Gulban O. F. De Martino F. Vu A. T. Yacoub E. Ugurbil K. Lenglet C. (2018). Cortical fibers orientation mapping using in-vivo whole brain 7T diffusion MRI. Neuroimage 178, 104–118. 10.1016/j.neuroimage.2018.05.010 29753105 PMC6118131

[B44] Hansen P. C. (1992). Analysis of discrete ill-posed problems by means of the L-curve. SIAM Rev. 34, 561–580. 10.1137/1034115

[B45] He Y. Aznar S. Siebner H. R. Dyrby T. B. (2021). In vivo tensor-valued diffusion MRI of focal demyelination in white and deep grey matter of rodents. Neuroimage 30, 102675. 10.1016/j.nicl.2021.102675 34215146 PMC8100629

[B46] Heidemann R. M. Porter D. A. Anwander A. Feiweier T. Heberlein K. Knösche T. R.. (2010). Diffusion imaging in humans at 7T using readout-segmented EPI and GRAPPA. Magn. Reson. Med. 64, 9–14. 10.1002/mrm.22480 20577977

[B47] Hendriks A. D. Luijten P. R. Klomp D. W. J. Petridou N. (2019). Potential acceleration performance of a 256-channel whole-brain receive array at 7 T. Magn. Reson. Med. 81, 1659–1670. 10.1002/mrm.27519 30257049 PMC6585755

[B48] Henriques R. N. Jespersen S. N. Shemesh N. (2020). Correlation tensor magnetic resonance imaging. Neuroimage 211, 116605. 10.1016/j.neuroimage.2020.116605 32044435

[B49] Huang S. Y. Witzel T. Keil B. Scholz A. Davids M. Dietz P.. (2021). Connectome 2.0: developing the next-generation ultra-high gradient strength human mri scanner for bridging studies of the micro-, meso- and macro-connectome. Neuroimage 243, 118530. 10.1016/j.neuroimage.2021.118530 34464739 PMC8863543

[B50] Jaermann T. De Zanche N. Staempfli P. Pruessmann K. Valavanis A. Boesiger P.. (2008). Preliminary experience with visualization of intracortical fibers by focused high-resolution diffusion tensor imaging. Am. J. Neuroradiol. 29, 146–150. 10.3174/ajnr.A0742 17947372 PMC8119111

[B51] Jensen J. H. Helpern J. A. Ramani A. Lu H. Kaczynski K. (2005). Diffusional kurtosis imaging: the quantification of non-gaussian water diffusion by means of magnetic resonance imaging. Magn. Reson. Med. 53, 1432–1440. 10.1002/mrm.20508 15906300

[B52] Jespersen S. N. (2012). Equivalence of double and single wave vector diffusion contrast at low diffusion weighting. NMR Biomed. 25, 813–818. 10.1002/nbm.1808 22134913

[B53] Jian B. Vemuri B. C. Özarslan E. Carney P. R. Mareci T. H. (2007). A novel tensor distribution model for the diffusion-weighted MR signal. Neuroimage 37, 164–176. 10.1016/j.neuroimage.2007.03.074 17570683 PMC2576290

[B54] Keil B. Blau J. N. Biber S. Hoecht P. Tountcheva V. Setsompop K.. (2013). A 64-channel 3T array coil for accelerated brain MRI. Magn. Reson. Med. 70, 248–258. 10.1002/mrm.24427 22851312 PMC3538896

[B55] Kellner E. Dhital B. Kiselev V. G. Reisert M. (2016). Gibbs-ringing artifact removal based on local subvoxel-shifts. Magn. Reson. Med. 76, 1574–1581. 10.1002/mrm.26054 26745823

[B56] Khan S. Vasung L. Marami B. Rollins C. K. Afacan O. Ortinau C. M.. (2019). Fetal brain growth portrayed by a spatiotemporal diffusion tensor MRI atlas computed from in utero images. Neuroimage 185, 593–608. 10.1016/j.neuroimage.2018.08.030 30172006 PMC6289660

[B57] Kim D. Doyle E. K. Wisnowski J. L. Kim J. H. Haldar J. P. (2017). Diffusion-relaxation correlation spectroscopic imaging: a multidimensional approach for probing microstructure. Magn. Reson. Med. 78, 2236–2249. 10.1002/mrm.26629 28317261 PMC5605406

[B58] Kingsbury B. F. (1920). The extent of the floor-plate of his and its significance. J. Compar. Neurol. 32, 113–135. 10.1002/cne.900320106

[B59] Kleinnijenhuis M. van Mourik T. Norris D. G. Ruiter D. J. van Cappellen van Walsum A. M. Barth M. (2015). Diffusion tensor characteristics of gyrencephaly using high resolution diffusion MRI in vivo at 7T. Neuroimage 109, 378–387. 10.1016/j.neuroimage.2015.01.001 25585019

[B60] Kleinnijenhuis M. Zerbi V. Küsters B. Slump C. H. Barth M. van Cappellen van Walsum A. M. (2013). Layer-specific diffusion weighted imaging in human primary visual cortex *in vitro*. Cortex 49, 2569–2582. 10.1016/j.cortex.2012.11.015 23347559

[B61] Koay C. G. Özarslan E. Johnson K. M. Meyerand M. E. (2012). Sparse and optimal acquisition design for diffusion mri and beyond. Med. Phys. 39, 2499–2511. 10.1118/1.3700166 22559620 PMC3344880

[B62] Koch M. A. Finsterbusch J. (2008). Compartment size estimation with double wave vector diffusion-weighted imaging. Magn. Reson. Med. 60, 90–101. 10.1002/mrm.21514 18421690

[B63] Komlosh M. Horkay F. Freidlin R. Nevo U. Assaf Y. Basser P. (2007). Detection of microscopic anisotropy in gray matter and in a novel tissue phantom using double pulsed gradient spin echo MR. J. Magn. Reson. 189, 38–45. 10.1016/j.jmr.2007.07.003 17869147

[B64] Komlosh M. E. Benjamini D. Hutchinson E. B. King S. Haber M. Avram A. V.. (2018). Using double pulsed-field gradient MRI to study tissue microstructure in traumatic brain injury (TBI). Microporous Mesoporous Mater. 269, 156–159. 10.1016/j.micromeso.2017.05.030 30337835 PMC6188654

[B65] Lampinen B. Zampeli A. Björkman-Burtscher I. M. Szczepankiewicz F. Källén K. Compagno Strandberg M.. (2020). Tensor-valued diffusion MRI differentiates cortex and white matter in malformations of cortical development associated with epilepsy. Epilepsia 61, 1701–1713. 10.1111/epi.16605 32667688 PMC7963222

[B66] Lasi,c S. Szczepankiewicz F. Eriksson S. Nilsson M. Topgaard D. (2014). Microanisotropy imaging: quantification of microscopic diffusion anisotropy and orientational order parameter by diffusion mri with magic-angle spinning of the q-vector. Front. Phys. 2, 11. 10.3389/fphy.2014.00011

[B67] Leergaard T. B. White N. S. de Crespigny A. Bolstad I. D'Arceuil H. Bjaalie J. G.. (2010). Quantitative histological validation of diffusion MRI fiber orientation distributions in the rat brain. PLoS ONE 5, e8595. 10.1371/journal.pone.0008595 20062822 PMC2802592

[B68] Lefévre J. Mangin J. F. (2010). A reaction-diffusion model of human brain development. PLoS Comput. Biol. 6, e1000749. 10.1371/journal.pcbi.1000749 20421989 PMC2858670

[B69] Leuze C. W. U. Anwander A. Bazin P. L. Dhital B. Stüber C. Reimann K.. (2014). Layer-specific intracortical connectivity revealed with diffusion MRI. Cereb. Cortex 24, 328–339. 10.1093/cercor/bhs311 23099298 PMC3888365

[B70] Lichtman J. W. Denk W. (2011). The big and the small: challenges of imaging the brain's circuits. Science 334, 618. 10.1126/science.1209168 22053041

[B71] Lin J. (1991). Divergence measures based on the shannon entropy. IEEE Trans. Inf. Theory 37, 145–151. 10.1109/18.61115

[B72] Liu C. Bammer R. Acar B. Moseley M. E. (2004). Characterizing non-gaussian diffusion by using generalized diffusion tensors. Magn. Reson. Med. 51, 924–937. 10.1002/mrm.20071 15122674

[B73] Lundell H. Nilsson M. Dyrby T. B. Parker G. J. M. Cristinacce P. L. H. Zhou F. L.. (2019). Multidimensional diffusion MRI with spectrally modulated gradients reveals unprecedented microstructural detail. Sci. Rep. 9, 9026. 10.1038/s41598-019-45235-7 31227745 PMC6588609

[B74] Magdoom K. N. Pajevic S. Dario G. Basser P. J. (2021). A new framework for MR diffusion tensor distribution. Sci. Rep. 11, 2766. 10.1038/s41598-021-81264-x 33531530 PMC7854653

[B75] McGivney D. Deshmane A. Jiang Y. Ma D. Badve C. Sloan A.. (2018). Bayesian estimation of multicomponent relaxation parameters in magnetic resonance fingerprinting. Magn. Reson. Med. 80, 159–170. 10.1002/mrm.27017 29159935 PMC5876128

[B76] McKinstry R. C. Mathur A. Miller J. H. Ozcan A. Snyder A. Z. Schefft G. L.. (2002). Radial organization of developing preterm human cerebral cortex revealed by non-invasive water diffusion anisotropy MRI. Cereb. Cortex 12, 1237–1243. 10.1093/cercor/12.12.1237 12427675

[B77] McNab J. A. Jbabdi S. Deoni S. C. L. Douaud G. Behrens T. E. J. Miller K. L. (2009). High resolution diffusion-weighted imaging in fixed human brain using diffusion-weighted steady state free precession. Neuroimage 46, 775–785. 10.1016/j.neuroimage.2009.01.008 19344686

[B78] McNab J. A. Polimeni J. R. Wang R. Augustinack J. C. Fujimoto K. Stevens A.. (2013). Surface-based analysis of diffusion orientation for identifying architectonic domains in the *in vivo* human cortex. Neuroimage 69, 87–100. 10.1016/j.neuroimage.2012.11.065 23247190 PMC3557597

[B79] Miller K. L. Stagg C. J. Douaud G. Jbabdi S. Smith S. M. Behrens T. E. J.. (2011). Diffusion imaging of whole, post-mortem human brains on a clinical MRI scanner. Neuroimage 57, 167–181. 10.1016/j.neuroimage.2011.03.070 21473920 PMC3115068

[B80] Mitra P. P. (1995). Multiple wave-vector extensions of the NMR pulsed-field-gradient spin-echo diffusion measurement. Phys. Rev. B 51, 15074. 10.1103/PhysRevB.51.15074 9978461

[B81] Mulkern R. V. Gudbjartsson H. Westin C.-F. Zengingonul H. P. Gartner W. Guttmann C. R. G.. (1999). Multi-component apparent diffusion coefficients in human brain? NMR Biomed. 12, 51–62. 10.1002/(SICI)1099-1492(199902)12:1<51::AID-NBM546>3.0.CO;2-E 10195330

[B82] Nagy Z. Alexander D. C. Thomas D. L. Weiskopf N. Sereno M. I. (2013). Using high angular resolution diffusion imaging data to discriminate cortical regions. PLoS ONE 8, e63842. 10.1371/journal.pone.0063842 23691102 PMC3656939

[B83] Nieuwenhuys R. (2013). The myeloarchitectonic studies on the human cerebral cortex of the Vogt-Vogt school, and their significance for the interpretation of functional neuroimaging data. Brain Struct. Funct. 218, 303–352. 10.1007/s00429-012-0460-z 23076375

[B84] Nilsson M. Lätt J. van Westen D. Brockstedt S. Lasič S. Ståhlberg F.. (2013). Noninvasive mapping of water diffusional exchange in the human brain using filter-exchange imaging. Magn. Reson. Med. 69, 1572–1580. 10.1002/mrm.24395 22837019

[B85] Nilsson M. Szczepankiewicz F. Brabec J. Taylor M. Westin C.-F. Golby A.. (2020). Tensor-valued diffusion mri in under 3 minutes: an initial survey of microscopic anisotropy and tissue heterogeneity in intracranial tumors. Magn. Reson. Med. 83, 608–620. 10.1002/mrm.27959 31517401 PMC6900060

[B86] Olman C. A. Harel N. Feinberg D. A. He S. Zhang P. Ugurbil K.. (2012). Layer-specific fMRI reflects different neuronal computations at different depths in human V1. PLoS ONE 7, e32536. 10.1371/journal.pone.0032536 22448223 PMC3308958

[B87] Özarslan E. Koay C. G. Shepherd T. M. Komlosh M. E. Irfanoglu M. O. Pierpaoli C.. (2013). Mean apparent propagator (MAP) MRI: a novel diffusion imaging method for mapping tissue microstructure. Neuroimage 78, 16–32. 10.1016/j.neuroimage.2013.04.016 23587694 PMC4059870

[B88] Özarslan E. Mareci T. H. (2003). Generalized diffusion tensor imaging and analytical relationships between diffusion tensor imaging and high angular resolution diffusion imaging. Magn. Reson. Med. 50, 955–965. 10.1002/mrm.10596 14587006

[B89] Pajevic S. Aldroubi A. Basser P. J. (2002). A continuous tensor field approximation of discrete dt-mri data for extracting microstructural and architectural features of tissue. J. Magn. Reson. 154, 85–100. 10.1006/jmre.2001.2452 11820830

[B90] Panagiotaki E. Schneider T. Siow B. Hall M. G. Lythgoe M. F. Alexander D. C. (2012). Compartment models of the diffusion mr signal in brain white matter: a taxonomy and comparison. Neuroimage 59, 2241–2254. 10.1016/j.neuroimage.2011.09.081 22001791

[B91] Pasternak O. Sochen N. Gur Y. Intrator N. Assaf Y. (2009). Free water elimination and mapping from diffusion MRI. Magn. Reson. Med. 62, 717–730. 10.1002/mrm.22055 19623619

[B92] Perrin M. Poupon C. Rieul B. Leroux P. Constantinesco A. Mangin J.-F.. (2005). Validation of Q-ball imaging with a diffusion fibre-crossing phantom on a clinical scanner. Philos. Trans. R. Soc. B Biol. Sci. 360, 881–891. 10.1098/rstb.2005.1650 PMC1854933 16087433

[B93] Petersen C. C. (2007). The functional organization of the barrel cortex. Neuron 56, 339–355. 10.1016/j.neuron.2007.09.017 17964250

[B94] Pierpaoli C. Basser P. J. (1996). Toward a quantitative assessment of diffusion anisotropy. Magn. Reson. Med. 36, 893–906. 10.1002/mrm.1910360612 8946355

[B95] Pierpaoli C. Walker L. Irfanoglu M. Barnett A. Basser P. Chang L.. (2010). Tortoise: an integrated software package for processing of diffusion mri data, in 18th Scientific Meeting of the International Society for Magnetic Resonance in Medicine (Stockholm), 1597.

[B96] Pirk C. M. Gómez P. A. Lipp I. Buonincontri G. Molina-Romero M. Sekuboyina A.. (2020). Deep learning-based parameter mapping for joint relaxation and diffusion tensor mr fingerprinting, in Medical Imaging With Deep Learning (Montreal, QC), 638–654.

[B97] Prange M. Song Y.-Q. (2009). Quantifying uncertainty in NMR T2 spectra using Monte Carlo inversion. J. Magn. Reson. 196, 54–60. 10.1016/j.jmr.2008.10.008 18952474

[B98] Reymbaut A. Critchley J. Durighel G. Sprenger T. Sughrue M. Bryskhe K.. (2021). Toward nonparametric diffusion- characterization of crossing fibers in the human brain. Magn. Reson. Med. 85, 2815–2827. 10.1002/mrm.28604 33301195 PMC7898694

[B99] Ronen I. Ercan E. Webb A. (2013). Axonal and glial microstructural information obtained with diffusion-weighted magnetic resonance spectroscopy at 7T. Front. Integr. Neurosci. 7, 13. 10.3389/fnint.2013.00013 23493316 PMC3595513

[B100] Rubenstein J. Rakic P. (2020). Neural Circuit and Cognitive Development: Comprehensive Developmental Neuroscience. Cambridge, MA: Academic Press.

[B101] Saleem K. S. Avram A. V. Glen D. Yen C. C.-C. Ye F. Q. Komlosh M.. (2021). High-resolution mapping and digital atlas of subcortical regions in the macaque monkey based on matched MAP-MRI and histology. Neuroimage 245, 118759. 10.1016/j.neuroimage.2021.118759 34838750 PMC8815330

[B102] Seehaus A. Roebroeck A. Bastiani M. Fonseca L. Bratzke H. Lori N.. (2015). Histological validation of high-resolution dti in human post mortem tissue. Front. Neuroanat. 9, 98. 10.3389/fnana.2015.00098 26257612 PMC4511840

[B103] Seehaus A. K. Roebroeck A. Chiry O. Kim D. S. Ronen I. Bratzke H.. (2013). Histological validation of DW-MRI tractography in human postmortem tissue. Cereb. Cortex 23, 442–450. 10.1093/cercor/bhs036 22345356 PMC3584953

[B104] Setsompop K. Fan Q. Stockmann J. Bilgic B. Huang S. Cauley S. F.. (2018). High-resolution in vivo diffusion imaging of the human brain with generalized slice dithered enhanced resolution: Simultaneous multislice (gSlider-SMS). Magn. Reson. Med. 79, 141–151. 10.1002/mrm.26653 28261904 PMC5585027

[B105] Shapson-Coe A. Januszewski M. Berger D. R. Pope A. Wu Y. Blakely T.. (2021). A connectomic study of a petascale fragment of human cerebral cortex. bioRxiv 2021.05.29.446289. 10.1101/2021.05.29.446289

[B106] Sjölund J. Szczepankiewicz F. Nilsson M. Topgaard D. Westin C.-F. Knutsson H. (2015). Constrained optimization of gradient waveforms for generalized diffusion encoding. J. Magn. Reson. 261, 157–168. 10.1016/j.jmr.2015.10.012 26583528 PMC4752208

[B107] Song Y. Ly I. Fan Q. Nummenmaa A. Martinez-Lage M. Curry W. T.. (2022). Measurement of full diffusion tensor distribution using high-gradient diffusion MRI and applications in diffuse gliomas. Front. Phys. 10, 813475. 10.3389/fphy.2022.813475

[B108] Stanisz G. J. Wright G. A. Henkelman R. M. Szafer A. (1997). An analytical model of restricted diffusion in bovine optic nerve. Magn. Reson. Med. 37, 103–111. 10.1002/mrm.1910370115 8978638

[B109] Stejskal E. O. Tanner J. E. (1965). Spin diffusion measurements: spin echoes in the presence of a time-dependent field gradient. J. Chem. Phys. 42, 288–292. 10.1063/1.1695690

[B110] Szczepankiewicz F. van Westen D. Englund E. Westin C.-F. Ståhlberg F. Lätt J.. (2016). The link between diffusion MRI and tumor heterogeneity: mapping cell eccentricity and density by diffusional variance decomposition (DIVIDE). Neuroimage 142, 522–532. 10.1016/j.neuroimage.2016.07.038 27450666 PMC5159287

[B111] Takahashi E. Dai G. Rosen G. D. Wang R. Ohki K. Folkerth R. D.. (2011). Developing neocortex organization and connectivity in cats revealed by direct correlation of diffusion tractography and histology. Cereb. Cortex 21, 200–211. 10.1093/cercor/bhq084 20494968 PMC3025725

[B112] Tax C. M. W. Dela Haije T. Fuster A. Westin C.-F. Viergever M. A. Florack L.. (2016). Sheet Probability Index (SPI): characterizing the geometrical organization ofthe white matter with diffusion MRI. Neuroimage 142, 260–279. 10.1016/j.neuroimage.2016.07.042 27456538 PMC5400114

[B113] Tax C. M. W. Westin C.-F. Dela Haije T. Fuster A. Viergever M. A. Calabrese E.. (2017). Quantifying the brain's sheet structure with normalized convolution. Med. Image Anal. 39, 162–177. 10.1016/j.media.2017.03.007 28511065 PMC5546268

[B114] Topgaard D. (2017). Multidimensional diffusion MRI. J. Magn. Reson. 275, 98–113. 10.1016/j.jmr.2016.12.007 28040623

[B115] Tournier J. Calamante F. Connelly A. (2012). Mrtrix: diffusion tractography in crossing fiber regions. Int. J. Imaging Syst. Technol. 22, 53–66. 10.1002/ima.22005

[B116] Tournier J. D. Calamante F. Gadian D. G. Connelly A. (2004). Direct estimation of the fiber orientation density function from diffusion-weighted mri data using spherical deconvolution. Neuroimage 23, 1176–1185. 10.1016/j.neuroimage.2004.07.037 15528117

[B117] Tuch D. S. Reese T. G. Wiegell M. R. Makris N. Belliveau J. W. Van Wedeen J. (2002). High angular resolution diffusion imaging reveals intravoxel white matter fiber heterogeneity. Magn. Reson. Med. 48, 577–582. 10.1002/mrm.10268 12353272

[B118] Turing A. M. (1952). The chemical basis of morphogenesis. Philos. Trans. R. Soc. Lond. B Biol. Sci. 237, 37–72. 10.1098/rstb.1952.0012 PMC4360114 25750229

[B119] Ugurbil K. Xu J. Auerbach E. J. Moeller S. Vu A. T. Duarte-Carvajalino J. M.. (2013). Pushing spatial and temporal resolution for functional and diffusion MRI in the Human Connectome Project. Neuroimage 80, 80–104. 10.1016/j.neuroimage.2013.05.012 23702417 PMC3740184

[B120] Van Essen D. C. (1997). A tension-based theory of morphogenesis and compact wiring in the central nervous system. Nature 385, 313–318. 10.1038/385313a0 9002514

[B121] Van Essen D. C. Drury H. A. (1997). Structural and functional analyses of human cerebral cortex using a surface-based atlas. J. Neurosci. 17, 7079–7102. 10.1523/JNEUROSCI.17-18-07079.1997 9278543 PMC6573261

[B122] Vasung L. Huang H. Jovanov-Milošević N. Pletikos M. Mori S. Kostovi,c I. (2010). Development of axonal pathways in the human fetal fronto-limbic brain: Histochemical characterization and diffusion tensor imaging. J. Anat. 217, 400–417. 10.1111/j.1469-7580.2010.01260.x 20609031 PMC2992416

[B123] Veraart J. Novikov D. S. Christiaens D. Ades-aron B. Sijbers J. Fieremans E. (2016). Denoising of diffusion MRI using random matrix theory. Neuroimage 142, 394–406. 10.1016/j.neuroimage.2016.08.016 27523449 PMC5159209

[B124] Vogt O. (1910). Die myeloarchitektonische Felderung des menschlichen Stirnhirns. J. Psychol. Neurol. 15, 221–232.

[B125] Wang F. Dong Z. Tian Q. Liao C. Fan Q. Hoge W. S.. (2021). In vivo human whole-brain Connectom diffusion MRI dataset at 760 *μ*m isotropic resolution. Sci. Data 8, 1–12. 10.1038/s41597-021-00904-z 33927203 PMC8084962

[B126] Wang Z. Bovik A. Sheikh H. Simoncelli E. (2004). Image quality assessment: from error visibility to structural similarity. IEEE Trans. Image Process. 13, 600–612. 10.1109/TIP.2003.819861 15376593

[B127] Wedeen V. J. Rosene D. L. Wang R. Dai G. Mortazavi F. Hagmann P.. (2012). The geometric structure of the brain fiber pathways. Science 335, 1628. 10.1126/science.1215280 22461612 PMC3773464

[B128] Westin C. F. Knutsson H. Pasternak O. Szczepankiewicz F. Özarslan E. van Westen D.. (2016). Q-space trajectory imaging for multidimensional diffusion MRI of the human brain. Neuroimage 135, 345–362. 10.1016/j.neuroimage.2016.02.039 26923372 PMC4916005

[B129] Yacoub E. Harel N. Ugurbil K. (2008). High-field fMRI unveils orientation columns in humans. Proc. Natl. Acad. Sci. U.S.A. 105, 10607–10612. 10.1073/pnas.0804110105 18641121 PMC2492463

[B130] Zhang H. Schneider T. Wheeler-Kingshott C. A. Alexander D. C. (2012). NODDI: Practical in vivo neurite orientation dispersion and density imaging of the human brain. Neuroimage 61, 1000–1016. 10.1016/j.neuroimage.2012.03.072 22484410

[B131] Zhang M.-Q. Zhou L. Deng Q.-F. Xie Y.-Y. Xiao T.-Q. Cao Y.-Z.. (2015). Ultra-high-resolution 3D digitalized imaging of the cerebral angioarchitecture in rats using synchrotron radiation. Sci. Rep. 5, 14982. 10.1038/srep14982 26443231 PMC4595735

