# Supplementary material for: COnstrained Reference frame diffusion TEnsor Correlation Spectroscopic (CORTECS) MRI: A practical framework for high-resolution diffusion tensor distribution imaging
Source: Front Neurosci. 2022 Dec 15;16:1054509. doi: 10.3389/fnins.2022.1054509 (PMC9798222; doi:10.3389/fnins.2022.1054509)
Supplement: Supplementary file 1 [file Data_Sheet_1.pdf]

The SNR-dependence of the 2D and 3D cDTD reconstruction accuracy

Figs. 3 and **4** in the manuscript illustrate the performance of the 3D and 2D CORTECS MRI reconstructions, respectively, as a function of SNR using Monte Carlo simulations. The ground truth spectra for the examples shown in **Figs. 3** and **4** are mixtures of three log-normal distributions with a wide range for diffusivity values aimed to illustrate complex microstructures that may be observed in cortical tissue. For both examples, we generated measurements using the same experimental design (b-values and gradient orientations) as in our fixed brain scans and analyzed simulated noisy data to reconstruct pieceweise continuous cDTDs using a non-negative linear least-squares method with L2-norm regularization (Hansen, 1992), as described in (Avram et al., 2019; Avram et al., 2021).

To quantify the reconstruction accuracy (i.e., potential biases as a function of SNR) we calculated three different similarity metrics between the estimated mean normalized and ground truth spectra at different SNR levels. Specifically we computed the mean squared error, structural similarity (Zhou et al., 2004), and the Jensen-Shannon Divergence (Lin, 1991). Note that the JSD is a symmetric measure of the relative information entropy between two distributions with value between 0 and ln(2). It shows a very similar dependence on SNR in both the 3D (**Fig. 3**) and 2D (**Fig. 4**) examples. While all these metrics suggest a similar SNR-dependence of the reconstruction accuracy, it should be noted that the plots in **Fig. S1** may change if we considered other ground truth distributions.

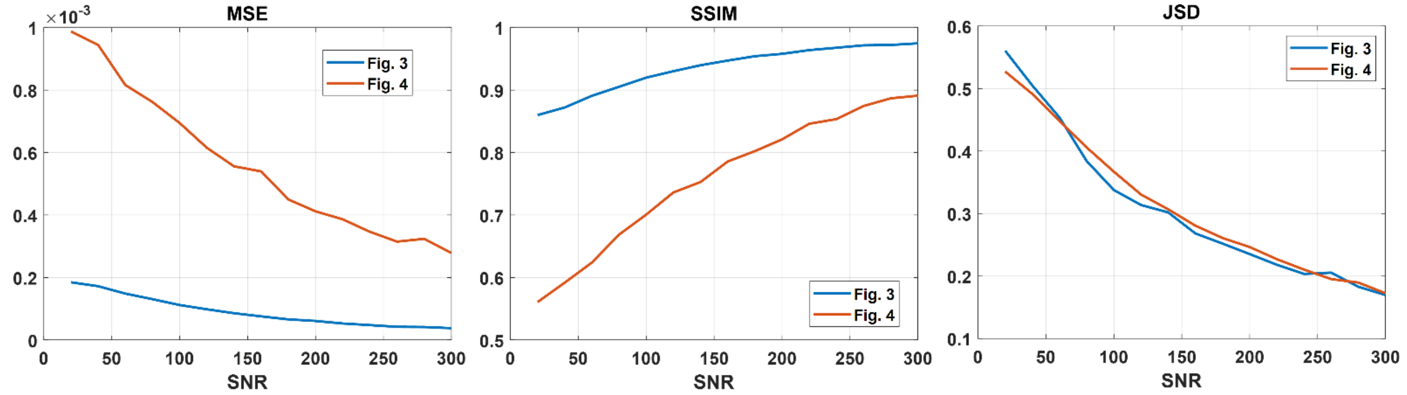

Supplementary Figure S1: Three metrics quantifying the similarity between the estimated mean normalized spectra and the corresponding ground truth distributions in **Fig. 3** (3D CORTECS MRI example) and **Fig. 4** (2D CORTECS MRI example). MSE – mean squared error; SSIM – structural similarity; JSD – Jensen-Shannon Divergence.

Statistical moments of the cDTD-derived micro FA and MD distributions

We derived distributions of the microscopic FA and MD parameters of the diffusion tensors comprising the non-parametric 2D cDTDs, $p(\lambda_r,\lambda_t)$, estimated in each voxel of the macaque cortex (**Fig. 6** in the manuscript). Specifically, we computed the non-parametric distributions $p_{FA}(\alpha)$ and $p_{MD}(\mu)$, respectively, where α and μ are random variables describing the microscopic FA and MD of the cDTD tensors. We quantified the means and variances of these distributions (**Fig. S2**) and visualized these important parameters in the same slice as shown in **Figs. 6** and **7**. The first moment (mean) of $p_{FA}(\alpha)$ quantifies the micro-FA, μFA, (Lasič et al., 2014; Magdoom et al., 2021; Topgaard, 2017; Westin et al., 2016) an important microstructural parameter in the dMRI literature.

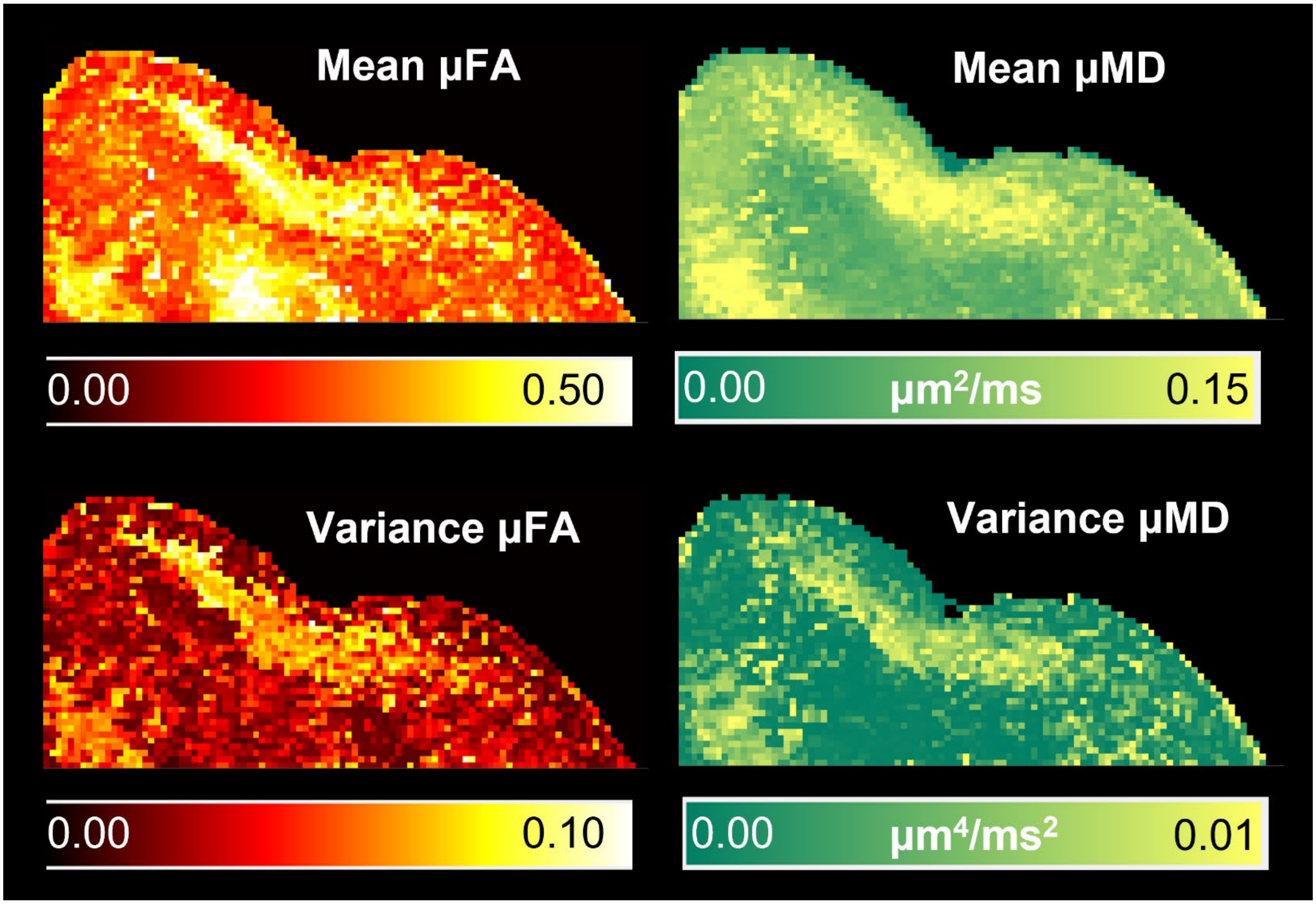

Supplementary Figure S2: From the 2D cDTDs estimated in the macaque monkey brain as the correlation spectra of principal diffusivities, λ_r and λ_t, along the radial and tangential orientations with respect to the cortical surface, respectively, (**Fig. 6** in the manuscript) we derived the distributions, $p_{FA}(\alpha)$ and $p_{MD}(\mu)$, of the FAs (left) and MDs (right) of the microscopic diffusion tensors, respectively, and computed their first (top row) and second (bottom row) statistical moments.

References

1. Avram, A.V., Sarlls, J.E., Basser, P.J., 2019. Measuring non-parametric distributions of intravoxel mean diffusivities using a clinical MRI scanner. Neuroimage 185, 255-262.
2. Avram, A.V., Sarlls, J.E., Basser, P.J., 2021. Whole-Brain Imaging of Subvoxel T1-Diffusion Correlation Spectra in Human Subjects. Frontiers in Neuroscience 15.
3. Hansen, P.C., 1992. Analysis of discrete ill-posed problems by means of the L-curve. SIAM review 34, 561-580.
4. Lasič, S., Szczepankiewicz, F., Eriksson, S., Nilsson, M., Topgaard, D., 2014. Microanisotropy imaging: quantification of microscopic diffusion anisotropy and orientational order parameter by diffusion MRI with magic-angle spinning of the q-vector. Frontiers in Physics 2.
5. Lin, J., 1991. Divergence measures based on the Shannon entropy. IEEE Transactions on Information Theory 37, 145-151.
6. Magdoom, K.N., Pajevic, S., Dario, G., Basser, P.J., 2021. A new framework for MR diffusion tensor distribution. Scientific Reports 11, 2766.
7. Topgaard, D., 2017. Multidimensional diffusion MRI. Journal of Magnetic Resonance 275, 98-113.
8. Westin, C.-F., Knutsson, H., Pasternak, O., Szczepankiewicz, F., Özarslan, E., van Westen, D., Mattisson, C., Bogren, M., O'Donnell, L.J., Kubicki, M., 2016. Q-space trajectory imaging for multidimensional diffusion MRI of the human brain. Neuroimage 135, 345-362.
9. Zhou, W., Bovik, A.C., Sheikh, H.R., Simoncelli, E.P., 2004. Image quality assessment: from error visibility to structural similarity. IEEE Transactions on Image Processing 13, 600-612.
